# Controllable Multinary Alloy Electrodeposition for Thin-Film Solar Cell Fabrication: A Case Study of Kesterite Cu_2_ZnSnS_4_

**DOI:** 10.1016/j.isci.2018.02.002

**Published:** 2018-03-08

**Authors:** Jie Ge, Yanfa Yan

**Affiliations:** 1 Department of Physics and Astronomy & Wright Center for Photovoltaics Innovation and Commercialization, The University of Toledo, Toledo, OH 43606, USA; 2 SNU Materials Division for Educating Creative Global Leaders, Seoul National University, Seoul 08826, Republic of Korea

**Keywords:** Energy Materials, Energy Sustainability, Materials Chemistry

## Abstract

Electrodeposition (ED) technology is a low-cost industrial candidate for solar cell fabrication. However, the practical aspects of controlling deposit morphology and composition have not been significantly addressed because of the complex co-plating variables that still need to be understood for multinary alloy ED. This work addresses these practical aspects on how to control composition and deposit morphology using co-electrodeposited kesterite alloy precursors as a case study. The alloy precursors co-plated under the optimized conditions from a mixed thiosulfate-sulfite electrolyte bath show uniform, smooth, and compact film morphology as well as uniform distribution of composition, well suited for efficient kesterite absorbers, finally delivering a Cu_2_ZnSnS_4_ (CZTS) thin-film solar cell with 7.4% efficiency based on a configuration Mo/CZTS/CdS/ZnO/aluminum-doped ZnO. This work underscores that alloy ED, with the advantage of controllable composition and morphology, holds promise for low-cost industrial manufacture of thin-film solar cells.

## Highlights

• Simultaneous control of the composition and morphology of the alloy electrodeposit
• Alloy electrodeposits with superb uniformity in film appearances and compositions
• The best kesterite Cu_2_ZnSnS_4_ mini solar cell with a 7.4% power conversion efficiency

## Graphical Abstract

**Figure:**
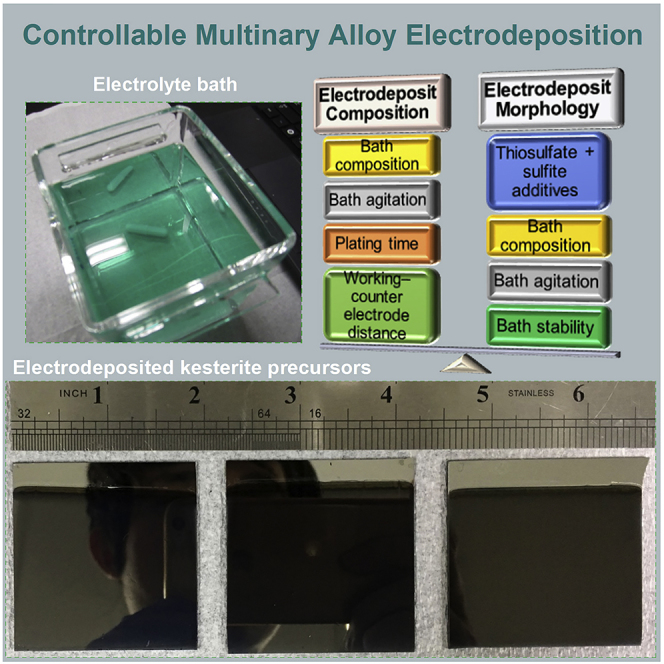

## Introduction

Electrodeposition (ED) is a non-vacuum and commercial technology with the demonstrated capability of depositing functional coatings on the base materials by using electric current to reduce dissolved salts in electrolyte baths. The feasibility of ED has been evaluated for the majority of the elements in the periodic table, including conventional metal elements (e.g., Cu, Zn, Sn, Au, Pt), alkaline earth metal elements (e.g., Mg [Amir et al., 2007], Sr [Huang, 1983], Eu [Gaikwad and Bhosale, 2001]), transition group metal elements (e.g., Nb [Giridhar et al., 2014], Ta [Krischok et al., 2013], Bi [Morris et al., 1954]), and non-metal elements (e.g., Se [Saji and Lee, 2013], Te [Alekperov, 1974]). Based on these elements, a variety of multifunctional coatings can be electrodeposited for applications of finishing, microelectronics, nanobiosystems, solar cell productions, etc. (Deligianni et al., 2011; Landolt, 2002, Schwarzacher et al., 2006). In particular, ED has met with success in preparing a wide range of photoelectrochemical and photovoltaic solar absorber films, including Sb_2_Se_3_ (Ngo et al., 2014), CdTe (Lincot, 2005), Cu_2_O (Dias et al., 2015), CuSbS_2_ (Rastogi and Janardhana, 2014, Septina et al., 2014), Cu(In, Ga)Se_2_ (CIGS) (Duchatelet et al., 2013, Lincot et al., 2004), Cu_2_ZnSnS(e)_4_ [CZTS(e)] (Colombara et al., 2015, Peter, 2015), and CH_3_NH_3_PbI_3_ (Chen et al., 2015, Huang et al., 2015). Aside from the absorbers, functional ZnO window layers (Tsin et al., 2015, Tsin et al., 2016a), Zn-based finger grids (Tsin et al., 2016b), and CuSCN (Ye et al., 2015) charge transport layers in the thin-film solar cells can be as well prepared by ED. Besides, ED has already demonstrated great success in the roll-to-roll CIGS solar panel manufacture, which is based on the precursor type of an electrodeposited Cu-In-Ga alloy covered by an electrodeposited In-Se or Ga-Se single layer (Aksu et al., 2012, Başol et al., 2009).

Kesterite CZTS(e) solar cells have attracted increasing attention in the past decade because of their non-toxicity and earth-abundant nature. ED emerging as a technoeconomic and large-scale deposition technology has been widely employed to fabricate the kesterite absorber precursors. Post-chalcogenization of the ED precursors is additionally needed to form the final kesterites. Promisingly, high-performance (7–9%) CZTS(e) solar cell devices have been frequently reported based on this two-stage approach (Guo et al., 2014, Jeon et al., 2014, Vauche et al., 2016). Specifically, electrodeposited precursors can be classified into two types: stack layers (i.e., Cu/Sn/Zn) and alloy layers (i.e., Cu-Zn-Sn), both of which have successfully attained the highly efficient kesterite devices. The device performances closely hinge on the quality of the kesterite absorber, which usually requires a Cu-poor and Zn-rich composition (Cu/Sn = 1.6–1.7, Cu/Zn ≤ 1.7, Zn/Sn ≥ 1.1), absence of secondary phases, and a large-area layer uniformity (Fairbrother et al., 2015, Tai et al., 2016, Vauche et al., 2016). Thus, it calls for ED with the ability to tune the precursor film composition and control its morphology sophisticatedly.

For the stacking ED precursor, the composition can be manipulated by varying the thickness of each individual layer. But the nucleation and uniformity of the overlayers largely depend on those of the underlying layers, and as a result, the stacking ED precursors usually fail to exhibit a smooth, uniform, and compact morphology (Scragg et al., 2010, Vauche et al., 2016). Meanwhile, the morphological inhomogeneity always comes with the compositional fluctuation. The direct chalcogenization annealing of stacked precursors usually results in poor film morphologies even with the formation of secondary phases, ultimately leading to poor device efficiencies (Lin et al., 2014, Scragg et al., 2010). The pre-mixing of the stacking ED precursors at moderately lower temperatures is known as one expedient to improve the compactness, morphology roughness, and atomic homogeneity, leading to an 8.2% efficiency using a kesterite selenide CZTSe absorber (Guo et al., 2014, Vauche et al., 2016). In contrast, the alloy ED precursor offers a tantalizing advantage over the stacking ED, making the pre-annealing of the precursors no longer needed before the chalcogenization, and an 8% efficiency has been achieved based on an alloy ED kesterite selenide CZTSe absorber (Jeon et al., 2014). Nonetheless, alloy ED seems more challenging in the practical aspects on how to control the alloy composition, largely because of the big difference between the reduction potentials of metal ions. Although the use of complexing reagents, such as low-cost citrate and tartrate salts (Abd El Rehim and El Ayashy, 1978, Gougaud et al., 2013, Guaus and Torrent-Burgués, 2005, Kazimierczak and Ozga, 2013, Lee et al., 2013, Mkawi et al., 2014, Shin et al., 2016, Slupska and Ozga, 2014), may minimize this potential difference, the reduction of Zn ion is rather negative and competes with the hydrogen evolution. To make the deposition of Zn more efficient, one expedient is to increase the deposition potential (or current density) (Abd El Rehim and El Ayashy, 1978, Chen et al., 2000, Gougaud et al., 2013, Kazimierczak and Ozga, 2013, Lee et al., 2013); worse, its side effect leads to hydrogen evolution parallel to the film deposition, particularly for acidic baths (pH < 5), and even leads to precursor films with a rough and dendritic morphology (Gougaud et al., 2013, Lee et al., 2013, Shin et al., 2016, Slupska and Ozga, 2014). As a result, few groups are able to obtain well-performing kesterite solar cells using alloy ED. Hitherto, none of the kesterite publications could succeed in refining electrodeposit compositions without sacrificing the deposit morphology. This may be due, largely, to the increased complexity of the ternary alloy ED that needs to handle with three different component metal elements, compared with the industrialized single or binary ED techniques.

The control of the alloy composition along with the deposit morphology needs the comprehensive and insightful knowledge of the co-plating process and the plating parameters, including bath compositions, plating potentials/current density, plating time, use of additives, and bath stability. This work addresses in detail these complicated operation variables of multinary alloy ED using kesterite CZTS as a case study. It is shown that the alloy composition and morphology can be exactly controlled through manipulation of these co-plating variables. Under the optimized conditions of alloy ED, the layer uniformity of precursors can rival those that were fabricated by the vacuum-based methods in the aspects of morphology and composition, manifesting the promise of alloy ED for the low-cost industrial fabrication of solar devices.

## Results and Discussion

### Plating Variables Optimization

The operation of plating variables is more complicated and critical to alloy ED than to depositions of single metals. The moderate changes in the plating parameters may alter the alloy composition and morphology considerably. All the critical co-plating variables are treated in this section, such as bath composition, agitation, plating time, distance between the working and counter electrodes, and the use of sodium thiosulfate (Na_2_S_2_O_3_) and sodium sulfite (Na_2_SO_3_) additives. We employed a conventional three-electrode assembly with a Ag/AgCl reference electrode, an inert Pt-coated Ti plate counter electrode, and a working electrode Mo-coated glass substrate for the investigation of the plating process and operation parameters. The electrolyte bath of 1 L contains 110 mM tri-sodium citrate (Na_3_C_6_H_5_O_7_) and 16 mM dipotassium tartrate (K_2_C_4_H_4_O_6_) as the complexing reagents. The depositions were carried out under the potentiostatic mode with a cathode potential of −1.135 volts versus Ag/AgCl (V_Ag/AgCl_).

#### Bath Composition and Agitation

In this subsection the effects of variations in the composition of the electrolyte bath on the composition and morphology of the electrodeposits are treated. As shown in Table 1, we find that the electrolyte based on a regular proportion of the initial metal salt content (recipe A1) only leads to a precursor with a heavily Zn-deficient composition and that increasing the concentration of Zn salt alone fails to increase the Zn content in the ED precursor considerably (recipe A2). This finding suggests that Cu and Sn species preferentially electrodeposit, since they have more positive reduction potentials than Zn species. Additionally, the precursors obtained from recipes A1 and A2 exhibit a gray color and a coarse appearance (high roughness). A detailed morphological characterization by the scanning electron microscope (SEM) suggests that the electrodeposits from recipes A1 and A2 consist of large ball-like crystals (1–2 μm) (Figures S1A and S1B). Usually, the surface morphology of electrodeposits hinges on the deposition rate, the degree of adsorption of impurities (i.e., brightening agents), and to a less extent on the overall set of the deposition conditions, such as the temperature, concentration of reducible species, nature of the reacting ions as well as the presence of foreign cations and anions, pH, and finally the character of the substrate. It is generally agreed that at a high deposition rate (rapid growth) coarse deposits with overgrown grains are formed. Thus, the coarse deposits obtained from recipes A1 and A2 indicate high deposition rates. Given that these two electrodeposits are severely deficient in Zn content, we therefore assume that the high deposit rates based on recipes A1 and A2 primarily come from rapid depositions of Cu and Sn species. The concentrations of Cu and Sn salts were then decreased (recipe A3), leading to not only a considerable Zn content increase in the electrodeposit (Table 1) but also reduced grain sizes (∼300-nm ball-like grains, see Figure S1C), compared with recipes A1 and A2. During the ED, the reducible species at the vicinity of the working electrode are consumed rapidly. The rest of the reducible ions in the bulk electrolyte therefore have to diffuse to the vicinity of the working electrode and replenish those ions consumed at the working electrode, that is, the so-called mass transport limitation (Schmidt and Gaida, 2017). For recipe A3, the decrease of Cu and Sn salts may lead to Cu and Sn deposits approaching to their mass-transport-limited values (Beattie and Dahn, 2005) that there are not sufficient Cu and Sn ions in the bath that can diffuse to the vicinity of the working electrode and replenish the consumed Cu and/or Sn ions right there. Under this condition, Zn is then preferentially deposited because of its very high bath concentration, and the Zn content in the deposit is thereby increased. However, the Zn content of the deposit from recipe A3 still falls short of the desirable stoichiometry for kesterites. Then, we further reduced the concentrations of Cu salt a little more by 2 mM and increased the Zn salt concentration largely by 10 mM (recipe A4). As shown in Table 1, the Zn composition obtained from recipe A4 was greatly increased, even though it is still a little poor compared with Sn (Sn/Zn = 0.93). We further increased the Zn salt concentration by 10 mM (recipe A5), achieving the desired Cu-poor and Zn-rich deposit. Besides, the deposits from recipes A4 and A5 demonstrate a black and mirror-like appearance and a fine-grained morphology with a grain size less than 40 nm (Figures S1D and S1E), which suggests that the deposition rate was effectively controlled by decreasing the concentrations of Cu and Sn salts down to certain levels in the electrolyte.

**Table 1:** Electrolyte Baths (1 L in Volume) with Various Metal Salt Concentrations and the Composition and Film Appearance of the Corresponding Electrodeposits

Electrolyte Bath						Electrodeposited Film^a^			
Recipe	Salt Concentration (mM)			Additive Concentration (mM)		Atomic Ratio			Appearance^b^
	CuSO_4_	SnSO_4_	ZnSO_4_	Na_2_S_2_O_3_	Na_2_SO_3_	Cu/Zn	Cu/Sn	Zn/Sn	
A1	15	10	10	5	1	12.20	1.83	0.15	Gray/coarse
A2	15	10	30	5	1	10.16	1.86	0.18	Gray/coarse
A3	9	6	30	5	1	2.80	1.79	0.64	Black/rough
A4	7	6	40	5	1	1.87	1.73	0.93	Black/mirror-like
A5	7	6	50	5	1	1.68	1.78	1.06	Black/mirror-like

a Note: the other plating parameters include (1) without agitation or bath heating, (2) 30 min plating time, and (3) 4 cm working-counter electrode distance.

b The defined degree of roughness: mirror-like (= extremely smooth) <smooth<rough<coarse (very rough).

In this subsection we obtained the best concentrations for the metal sulfate salts, which are 7 mM for Cu, 6 mM for Sn, and 50 mM for Zn. Under these chemical concentrations, the reduction of Cu and Sn ions are at their diffusion-limited rates, and then the Zn ions are preferentially deposited because of its very high concentration. It should be pointed out that all the electrodepositions herein and hereafter are carried out without stirring the electrolyte. Instead, even a slow agitation can break up the diffusion limitations for Cu and Sn ions because it promotes bulk transporting of the Cu and Sn ions in the electrolyte to the vicinity of the working electrode much faster. Under agitation, the overall deposition rate is therefore increased and the onset of Zn preferential deposition is limited. As a consequence, the electrodeposit is deficient in Zn and the surface roughness and grain size increase (see Figure S2).

#### Plating Time

**Figure 1:**
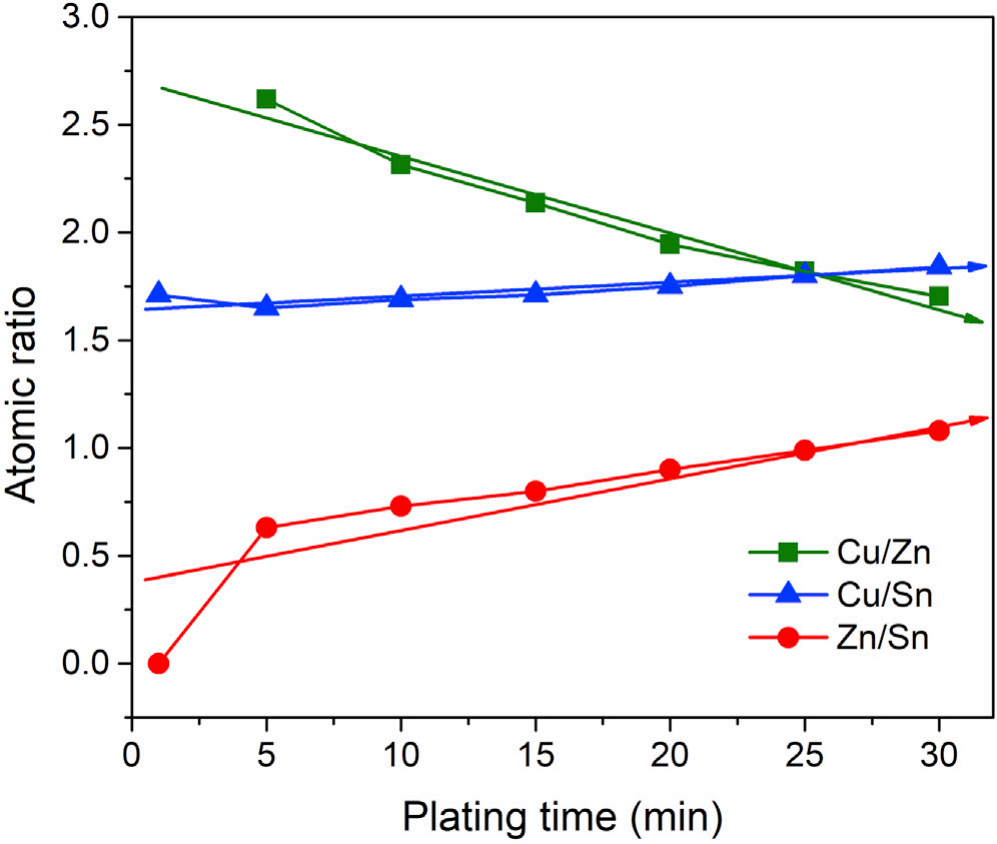
The Element Atomic Ratios of the Electrodeposits Using Various Plating Time Note: electrolyte bath (1 L in volume) based on recipe A5; the other plating parameters include (1) without agitation or bath heating and (2) 4 cm working-counter electrode distance.

Since the composition of electrodeposits can be tuned by mass transport control, the deposit composition may vary with the electroplating time. To verify this point, we prepared a series of precursors on Mo substrates with different electroplating time durations from 1 to 30 min using recipe A5. As shown in Figure 1, we find that the electrodeposits grown with the shorter plating time exhibit Zn-poor compositions and that the Zn contents in the deposits become richer as the plating time increases. This suggests that Cu and Sn are preferentially deposited at the very beginning of the electrodeposition. As the Cu and Sn ions at the vicinity of the working electrode are gradually consumed, the deposition of Zn ions is then triggered. For a 30-min electrodeposit, a Zn content gradient in depth should exist, whereby Zn is rich at the regions close to the film top but poor at the film rear side close to the Mo substrate. Figure S3 additionally shows the top-view SEM images at high magnifications of the electrodeposits with the plating timings varying from 1 to 30 min. We find that all the electrodeposits exhibit the fine-grained crystalline nature with the grain size less than ∼40 nm, suggesting a high nucleation rate and a uniform nucleation/growth process during the entire deposition.

#### Distance between Working and Counter Electrodes

**Figure 2:**
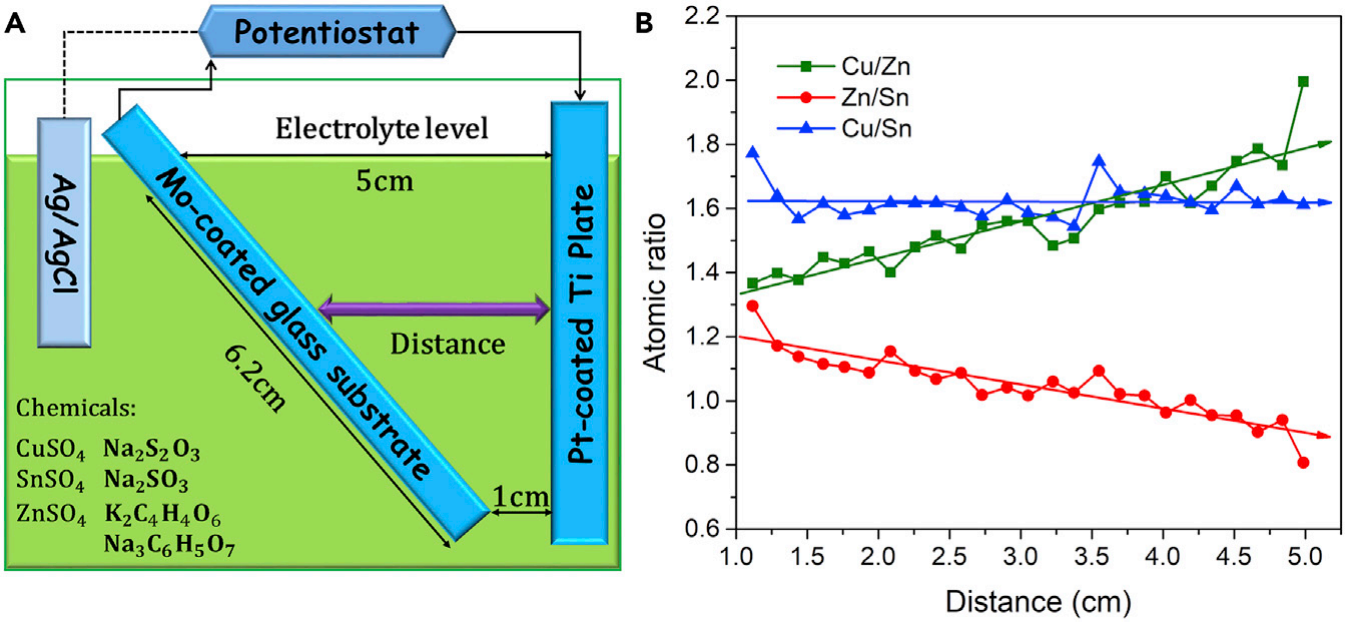
Hull Cell Test Showing the Effects of the Variation of the Working-Counter Electrode Distance on Compositions of the Electrodeposits (A) Schematic drawing for Hull cell set-up. (B) Atomic ratios of the electrodeposit versus the studied working-counter electrode distance. Note: electrolyte bath (1 L in volume) based on recipe A5; the other plating parameters include (1) without agitation or bath heating and (2) 30 min plating time.

In this subsection, we investigate how the working-counter electrode distance influences the composition of the alloy electrodeposits. We devised a method by which a composition spread library of alloys could be deposited with continuously varying working-counter electrode distances, that is, the Hull cell set-up (Figures 2A and S4A). In the Hull cell (Beattie and Dahn, 2005), the working electrode slants at a certain angle, and thereby it is no longer parallel with or equidistant from the counter electrode. This geometry can lead to the distance between the working and counter electrodes to gradually vary from 1 to 5 cm and thus establish a current density gradient along the length of the working electrode (Figure 2A): the closer the working and counter electrodes are the higher the current density between them will be. We measured the element composition distribution along the length of the electrodeposit with a size of 2.5 cm (width) by 5 cm (length) obtained from this set-up (see Figure S4A, where we measured the film thickness and composition along the labeled lines). As shown in Figure 2B, we find that the Cu/Sn ratio does not vary significantly, whereas the Cu/Zn and Sn/Zn ratios increase linearly from the high-current-density end (i.e., the small working-counter electrode distance) to the low-current-density end (i.e., the large working-counter electrode distance), suggesting a Zn gradient along the length of the working electrode that is Zn richer at the high-current-density end and poorer at the low-current-density end. Actually, the observation of this Zn gradient is also related to the mass-transport deposition mechanism. At the low-current-density end, the Sn and Cu ions around the working electrode are consumed slowly; hence, the Sn and Cu ions in the electrolyte bulk may have enough time to diffuse in and replenish the consumed Sn and Cu ions around the working electrode. As a result, the preferential deposition of Zn at the low-current-density end is alleviated or retarded. Hence, Cu and Sn are relatively richer at the low-current-density end. At the high-current-density end, the Sn and Cu ions around the working electrode are consumed rapidly; therefore, the concentration difference between Zn and Cu and Sn ions becomes more significant, favoring the following deposition of Zn. Thus, decreasing the working-counter electrode distance, which amounts to increasing the current density (i.e., deposition potential), can increase the Zn content in the electrodeposits. Aside from the Zn gradient, a gradient of layer thickness has also been established by the Hull set-up, which gradually increases from ∼685 nm at the low-current-density end to ∼765 nm at the high-current-density end (Figure S4B). Through the Hull cell tests, we obtained the best working-counter electrode distance, 4 cm, for the electrodeposition of kesterite precursors.

#### Thiosulfate and Sulfite based Additives

To obtain bright deposits, inorganic and organic additives are widely employed in the electroplating technology, which function as brighteners, inhibitors, and leveling agents and may complex with the metal ions, impede the reduction/nucleation of reducible metal ions as an interfacial adsorbate, make the film growth rate uniform and coherent, and hinder the overall growth of grains. The selection of the appropriate additive is very critical to the plating system. In this work, we use small amounts of sodium thiosulfate (5 mM) and sodium sulfite (1 mM) additives in our best electrolyte chemical bath for the kesterite precursor deposition. The actual functions of these two additives are revealed below.

**Figure 3:**
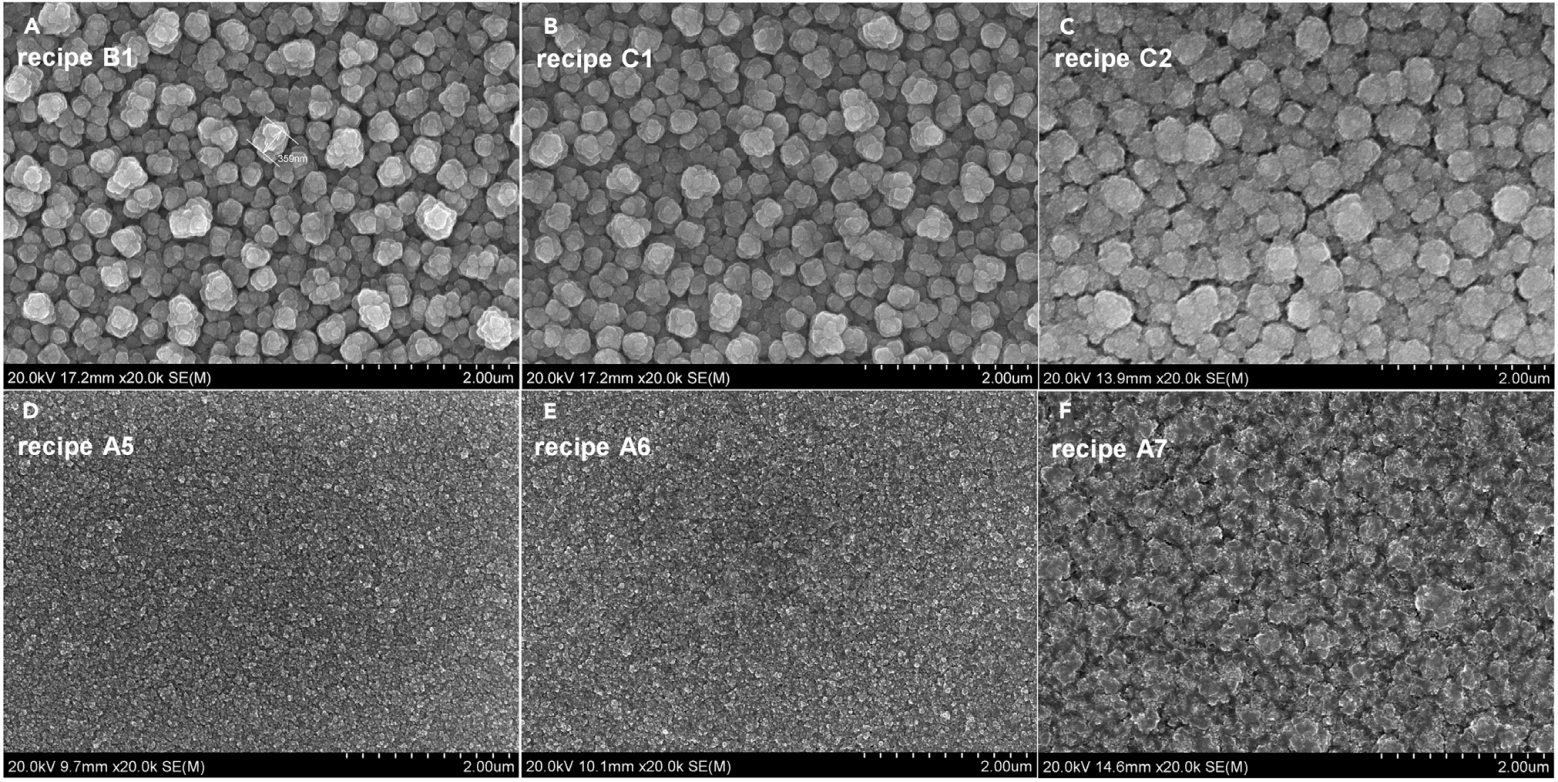
Top-View SEM Images of the Electrodeposits with Nearly Stoichiometric Metal Compositions Desirable for Kesterite Absorbers, Deposited Using the Electrolyte Baths (1 L in Volume) as Described in Table 2 (A) Recipe B1; (B) recipe C1; (C) recipe C2; (D) recipe A5; (E) recipe A6; (F) recipe A7. Note: the other plating parameters include (1) without agitation or bath heating, (2) 30 min plating time, and (3) 4 cm working-counter electrode distance.

First of all, with the knowledge and experience accumulated in the previous subsections, we obtained several different chemical recipes without the use of the sodium thiosulfate additive, such as recipes B1, C1, and C2 in Table 2, all of which can lead to the nearly stoichiometric metal composition required for kesterites. The electrodeposits from these three recipes exhibit the silver or silver-gray color and rough appearances (Figure S5), in contrast to the black color and mirror-like appearance of the deposits from the sodium thiosulfate-containing recipe (Figure S4A inset). The top-view SEM images shown in Figures 3A–3C and the cross-sectional SEM images shown in Figures S6A–S6C indicate large grain sizes of 300–500 nm and the rough morphologies, similar to the deposit morphology from the sodium thiosulfate-free electrolyte widely reported in the literature (Araki et al., 2009, Ennaoui et al., 2009, Juškėnas et al., 2012). These deposits were then annealed at 320°C for 30 min in a pure argon environment. Indeed, the pre-annealing promotes the re-crystallization of the deposits and makes the film much more compact; however, the surface roughness has not been considerably reduced (see Figures S6D–S6F).

**Table 2:** Electrolyte Baths (1 L in Volume) and Their Corresponding Electrodeposits Showing the Functions of Sodium Thiosulfate/Sulfite Additives

Electrolyte Bath							Electrodeposited Film^a^
Recipe	Salt Concentration (mM)			Additive Concentration (mM)		Bath Color; Stability	Appearance^b^
	CuSO_4_	SnSO_4_	ZnSO_4_	Na_2_S_2_O_3_	Na_2_SO_3_		
B1	7	6	31	0	0	Sky-blue; 2∼4 days	Silver/rough
C1	7	6	31	0	1	Sky-blue; 2∼4 days	Silver gray/rough
C2	7	6	32	0	5	Sky-blue; 2∼4 days	Gray/rough
A5	7	6	50	5	1	Greenish blue; 1<stability<2 days	Black/mirror-like
A6	7	6	50	5	0	Greenish blue; 0.5<stability<1 day	Black/mirror-like
A7	7	6	41	5	5	Greenish blue; 1<stability<2 days	Black/smooth

a Note: the electrodeposits exhibit nearly stoichiometric metal compositions that are required for kesterite absorbers; the other plating parameters include (1) without agitation or bath heating, (2) 30 min plating time, and (3) 4 cm working-counter electrode distance.

b The defined degree of roughness: mirror-like (= extremely smooth) <smooth<rough<coarse (= very rough).

On the other hand, the sodium thiosulfate (5 mM)-containing electrolyte baths (recipes A5 and A6) led to fine-grained deposits (Figures 3D and 3E) with mirror-like appearances. These distinct observations in deposit morphology and appearance suggest that sodium thiosulfate can function as a brightener and leveling agent. At the working electrode, thiosulfate ions give to the electrochemical reaction

(Equation 1) S_2_O_3_^2–^ (aq.) + H^+^ (aq.) + 2e^–^ ↔ S^2–^ + HSO_3_^–^ (aq.),

such that the reduction of thiosulfate ions releases sulfur to form metal sulfides in the deposits (Yukawa et al., 1996). Thus, this additional electrochemical reaction between sulfur and metal ions at the working electrode takes part in controlling the deposition rates and making the growth rate coherent, averting the grain overgrowth along certain orientations. We additionally find that the Zn salt concentrations for the electrolyte baths containing sodium thiosulfate are 10–20 mM larger than those in the electrolyte baths without sodium thiosulfate (Table 2), which suggests that sodium thiosulfate may greatly impede the deposition of Zn under the selected plating conditions. As a result, higher concentrations of Zn salts were used because of the presence of sodium thiosulfate.

**Figure 4:**
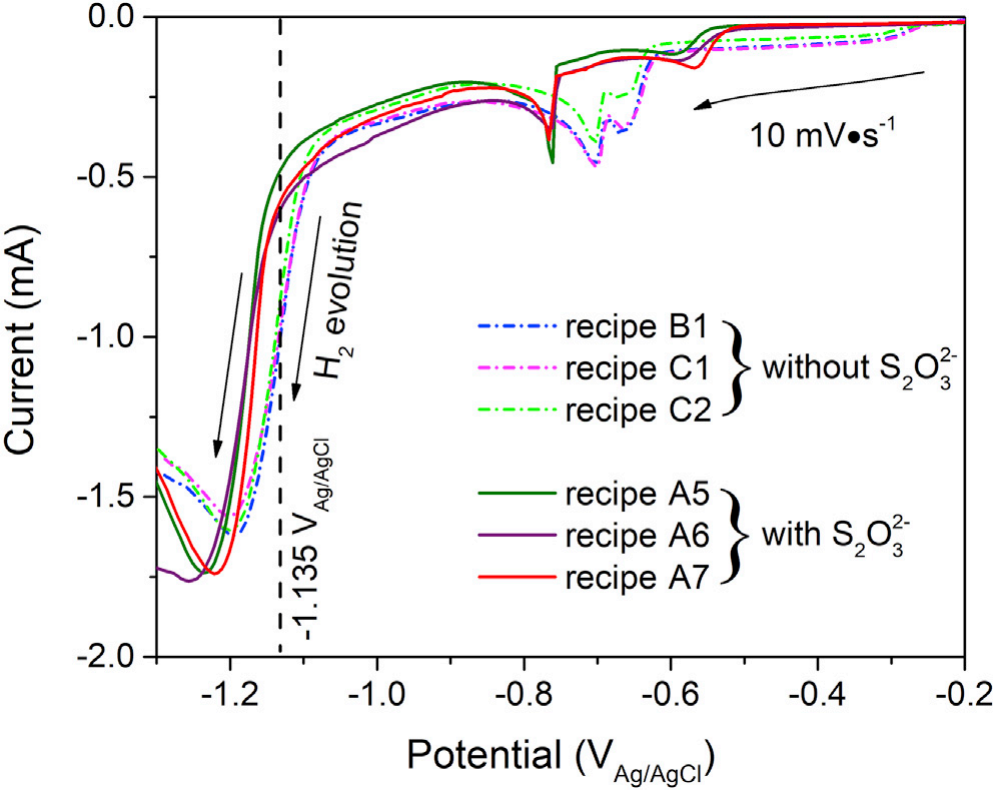
Linear Sweep Voltammetry Scans of the Different Electrolyte Baths (See Table 2) with and without Sodium Thiosulfate Additive

We carried out the linear sweep voltammetry (LSV) measurements of the electrolyte baths as shown in Figure 4. During the tests, it can be seen that hydrogen bubbles evolved off the working electrode (fresh Mo substrate) surface when the applied cathode potentials went more negatively than −1.10 to –1.15 V_Ag/AgCl_. The onsets of the hydrogen evolution reactions for the electrolyte baths without sodium thiosulfate occurred at around −1.10 V_Ag/AgCl_, whereas the onsets were shifted more negatively to about −1.15 V_Ag/AgCl_ for the electrolyte baths containing sodium thiosulfate (5 mM). During the regular deposition processes driven by the cathode potential of −1.135 V_Ag/AgCl_, a handful of generated hydrogen bubbles usually adheres to the surfaces of the deposits from the electrolyte baths without sodium thiosulfate additive, the adhesion of which leads to undesirable pits at the plated surfaces as seen by the top-view SEM imaging at low magnifications (Figures S7A–S7C). In contrast, no hydrogen evolution was seen for the electrolyte baths containing sodium thiosulfate, and its electrodeposit exhibited large-scale uniformity without any macroscopic point defects at the surface (Figures S7D–S7F). All these observations suggest that the sodium thiosulfate additive can act as an effective inhibitor against hydrogen evolution. At the working electrode, thiosulfate ions take part in the electrochemical reaction (1), meaning that the reduction of thiosulfate ions consumes protons around the working electrode. As a result, few protons are left at the vicinity of the working electrode and the odds of hydrogen evolution can be decreased, even thoroughly averted.

We additionally observed that the electrolyte baths without the sodium thiosulfate additive exhibited a sky-blue color due to Cu^2+^ ions (Figure S8). However, the sky-blue color gradually faded after sodium thiosulfate (5 mM) was added into the baths, and the electrolyte baths finally turned to greenish blue after about 15 min agitation. The fading away of the characteristic sky-blue color of Cu^2+^ suggests the reduced concentration of Cu^2+^ after the addition of sodium thiosulfate. Actually, the thiosulfate ion is a strong reductive agent, which can reduce Cu^2+^ to colorless Cu^+^, 2S_2_O_3_^2–^ (aq.) + 2Cu^2+^ (aq.) ↔ 2Cu^+^ (aq.) + S_4_O_6_^2–^ (aq.), and complex the Cu^+^, 2Cu^+^ (aq.) + 2S_2_O_3_^2–^ (aq.) ↔ Cu_2_(S_2_O_3_)_2_^2–^ (aq.) (Tykodi, 1990). Also, the use of sodium thiosulfate additive has caused apparent changes of the reduction potentials of the reducible species in the baths (see the LSV curves at the potential range from –0.25 to –0.85 V_Ag/AgCl_ in Figure 4). This indicates different redox reactions between the baths with and without sodium thiosulfate additive, in line with the phenomenon of bath color changes before and after the addition of sodium thiosulfate (Figure S8).

**Figure 5:**
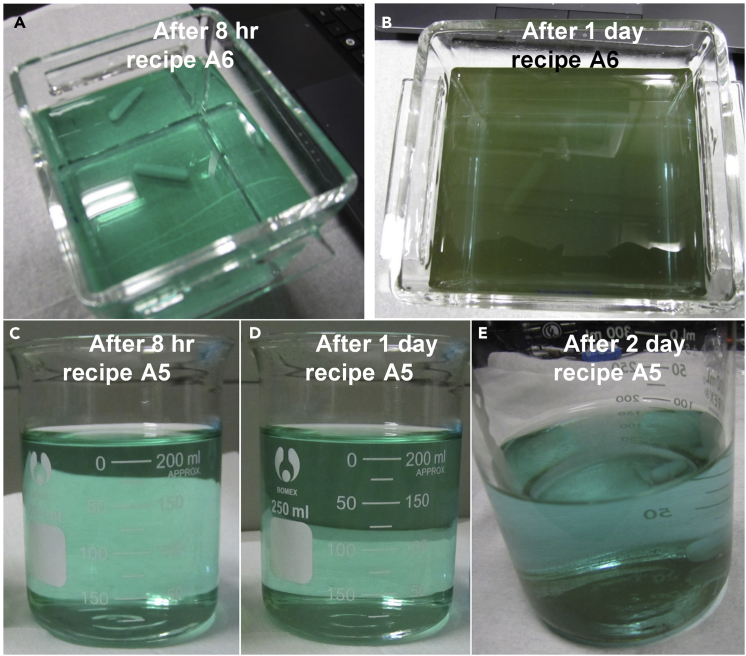
Photos of the Best Two Electrolyte Baths Containing 5 mM Sodium Thiosulfate Additive after Different Storage Time in Air, Showing that 1 mM Sodium Sulfite Additive Can Considerably Improve the Bath Stability (A) Recipe A6 after 8 hr; (B) recipe A6 after 1 day; (C) recipe A5 after 8 hr; (D) recipe A5 after 1 day; (E) recipe A5 after 2 days.

Concerning the bath stability, it should be pointed out that thiosulfate ion can release sulfur colloidal under acidic and neutral conditions, due to its disproportionation reaction,

(Equation 2) S_2_O_3_^2–^ (aq.) ↔ S + SO_3_^2–^ (aq.),

hence, thiosulfate is only relatively stable at low concentration and high pH (Green, 2007). In this regard, we chose a low concentration, 5 mM, of sodium sulfate in this treatise. As shown in Figure 5A, the electrolyte bath (recipe A6) containing 5 mM sodium thiosulfate alone can be stable for at least 8 hr without any visible bath degradations; however, after 1 day, the electrolyte became opaque and lots of dark yellow (nearly black) precipitates appeared (Figure 5B). With the addition of 1 mM sodium sulfite, the electrolyte bath (recipe A5) showed the significantly improved stability as long as 1 day (Figure 5D). A little excess sulfite in the electrolyte tends to drive the disproportionation reaction (2) to the left side and thereby improve the bath stability (Green, 2007). Besides, the addition of a little bit of sodium sulfite (1 mM) did not considerably change the composition and surface morphology of the electrodeposits (Figures 3D and 3E). After two days' storage, the precipitates appeared in the electrolyte bath of recipe A5 (Figure 3E). More sodium sulfite salts (5 mM) were then used (recipe A7); however, the bath stability did not seem to improve (Figure S9) compared with that of recipe A6. Worse, a rougher film surface with bumps (Figure 3F) has become of the electrodeposit from recipe A7, possibly owing to the presence of more sulfite ions, which can suppress thiosulfate ions to release sulfur by driving the electrochemical reaction (1) to the left. Herein, 1 mM sodium sulfite suffices to sustain a satisfactory bath stability (>1 day) without sacrificing the surface smoothness of the electrodeposits.

For the electrolyte baths without sodium thiosulfate (recipes B1, C1, and C2), they indeed show substantially better stabilities (2–3 days) than those containing sodium thiosulfate (recipes A5, A6, and A7). Nonetheless, these baths without sodium thiosulfate additive still demonstrate not-so-lasting stabilities, with black precipitates appearing after 3–4 days' storage (Figure S10), whereas the bath colors remain unchanged. We additionally measured the energy dispersive X-ray (EDX) composition of these filtered black precipitates (Figure S11). We find that these precipitates from the baths without thiosulfate are primarily made of Cu along with a tiny amount of Sn, whereas those from the baths containing thiosulfate are mainly made of Cu and sulfur concurrent with tiny amounts of Sn and potassium. The instability issues appearing in the baths with/without thiosulfate additive may largely be akin to the stability constants of the copper/tin-related complexes (Nunes, 1970).

### Precursor Characterization

The preceding section has dealt with the effects of plating variables, such as bath composition, agitation, plating time, and working-counter electrode distance, on the composition and morphology of alloy deposits and the roles that thiosulfate and sulfite additives play in the electrolyte bath. After extensive optimization studies, we have obtained the best deposition conditions for device-grade kesterite precursor depositions as follows:

Electrolyte bath (recipe A5, 1 L): trisodium citrate (110 mM), dipotassium tartrate (16 mM), tin sulfate (6 mM), zinc sulfate (50 mM), copper sulfate (7 mM), sodium thiosulfate (5 mM), sodium sulfite (1 mM), and highly purified water (1 L).

Plating parameters: no agitation during the deposition, plating time (30 min), working-counter electrode distance (4 cm), cathode potential (–1.135 V_Ag/AgCl_ for Mo substrate, −1.145 V_Ag/AgCl_ for indium tin oxide [ITO] substrate, and −1.16 V_Ag/AgCl_ for fluorine-doped SnO_2_ [FTO] substrate, where the depositions on ITO and FTO need slightly more negative cathode potentials to compensate the potential drops on the substrates due to the limited conductivities of themselves compared with the highly conductive Mo substrate). Before the electrodeposition of the precursor film, the fresh electrolyte was passed through by a low current for the necessary timings, which is referred to as pre-electrolysis (or dummying).

**Figure 6:**
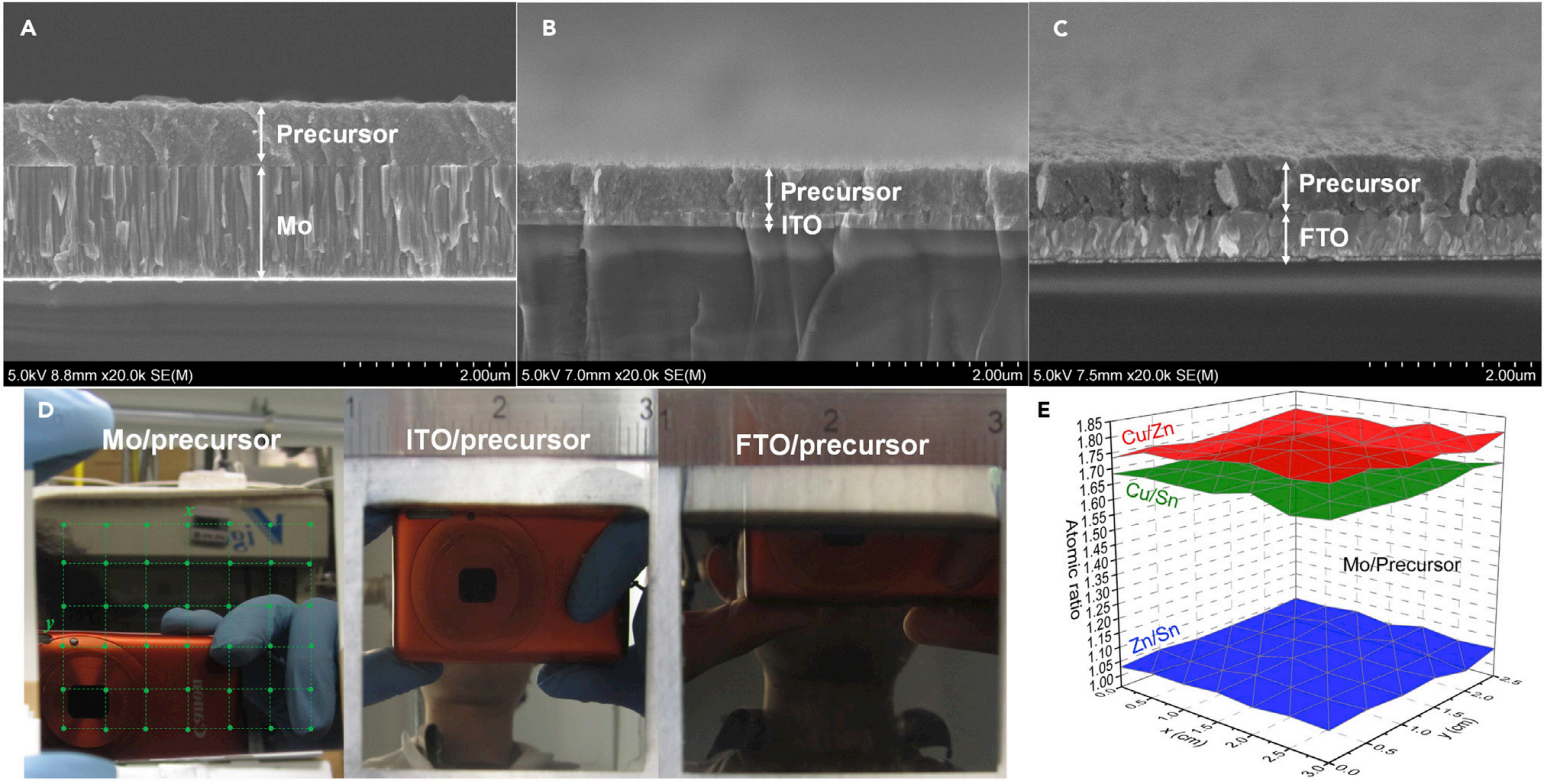
Uniformity Evaluation of Electrodeposited Precursors on Mo, ITO, and FTO Substrates (2 by 2 inches) Cross-sectional SEM images of the precursor films on (A) Mo, (B) ITO, and (C) FTO substrates; (D) the photos of these precursors showing the uniform, bright, and mirror-like film appearances; (E) metal element atomic ratios from the boxed area (x=3 cm by y=2.5 cm, see panel [D]) showing the element distribution of the precursor based on Mo substrate.

Figure 6 shows the cross-sectional SEM images of the electrodeposited precursors based on the Mo, ITO, and FTO substrates. All the obtained precursors are uniform, extremely smooth, very compact, and free from pin holes, comparable with the vacuum-deposited counterparts. No significant grain boundary is visible, again indicating the nanocrystalline fine-grained feature. Besides, we find that there is no obvious difference in morphology and crystalline characteristic among the precursors based on these three different substrates, suggesting that deposits are determined only by the deposition conditions. The photos of these precursor films (Figure 6D) on 2 by 2-inch substrates show the uniform, bright, and mirror-like appearances. Bright deposits usually take place when its grain size is much smaller than the visible light wavelength, 400 nm. To evaluate the element distribution, we conducted EDX spectroscopy tests on 42 different locations (see the green dots in Figure 6D) from a 3 × 2.5 cm^2^ area of an electrodeposit precursor based on the Mo substrate, with the compositional results being presented in Figure 6E and Table S1. As seen, the precursor on Mo exhibits a uniform distribution of constituent Cu, Zn, and Sn elements within the area studied, the ratios of which (Cu/Zn = 1.66–1.71, Zn/Sn = 1.03–1.07, and Cu/Sn = 1.73–1.80) are well suitable for the highly efficient kesterite absorbers.

**Figure 7:**
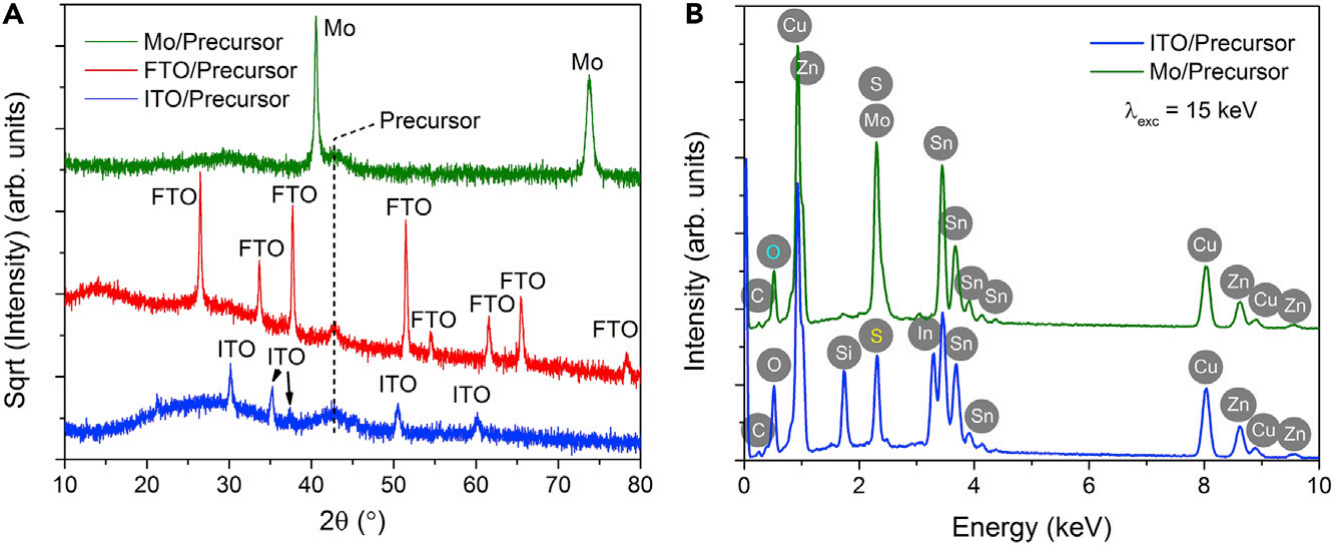
Structural and Compositional Studies of Electrodeposited Precursor Films on Mo, ITO, and FTO Subtrates (A) X-ray diffraction (XRD) patterns; (B) energy dispersive X-ray (EDX) spectra.

To gain comprehensive knowledge on the aspects of the constituent phases and chemical elements in the electrodeposited alloy precursor coatings, we performed further examinations by means of X-ray diffraction (XRD), EDX spectra, and X-ray photoemission spectroscopy (XPS). As shown in Figure 7A, the XRD patterns yield no apparent diffraction lines coming from the precursor coatings but only a weak and broad peak observed at 2*θ* ≈ 43°, suggesting a characteristic of nanocrystalline fine-grained structure, consistent with the SEM observations in Figures 3E and 6. As seen in the EDX profiles in Figure 7B, the precursor film on Mo substrate demonstrates an apparently intense peak at ∼0.51 keV from oxygen. The observed oxygen may not possibly originate from the glass substrate, because (1) there is no apparent silicon signal from the glass and (2) the electron beam for the EDX analysis accelerated by a 15 kV voltage cannot penetrate through the entire precursor together with the 1.5-μm-thick Mo compact layer to reach the glass substrate underneath (Ishizuka et al., 2014). Thus, it can be confirmed that the precursor layer contains a large amount of oxygen. We additionally find the evidence of carbon present in the precursor from the EDX profiles. Owing to the overlap of the EDX signals of Mo and S, we have to quantify the sulfur composition using the EDX data of the precursor grown on ITO and FTO substrates: S/Zn ≈ 0.89 and S/Cu ≈ 0.5, where carbon and oxygen were neglected because the EDX quantification for these light elements is not reliable.

**Figure 8:**
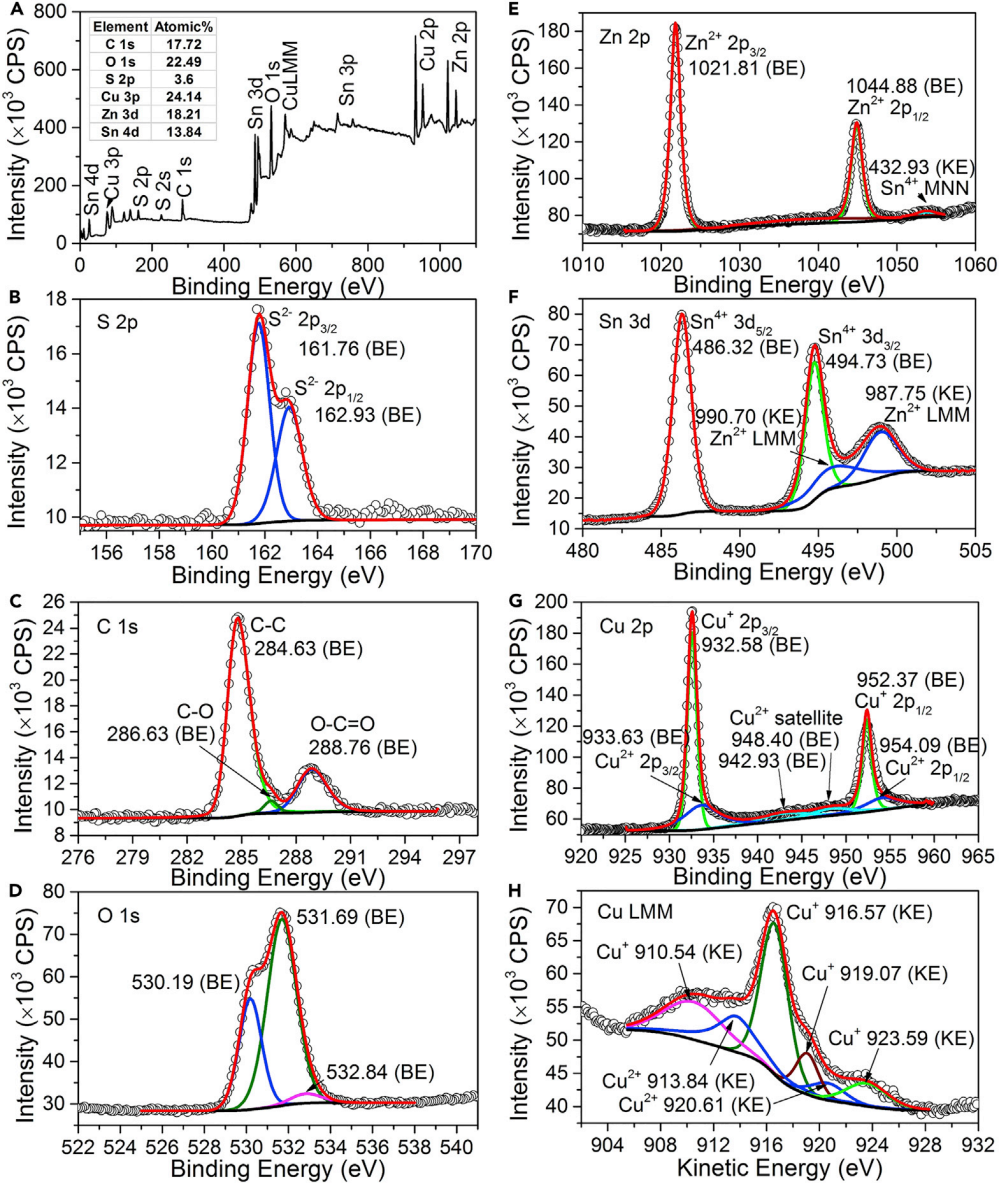
X-ray Photoelectron Spectroscopic (XPS) Measurements of An Electrodeposited Precursor Film (A) Full survey spectrum and element quantification result; (B-H) detailed measurements at the region of (B) S 2p, (C) C 1s, (D) O 1s, (E) Zn 2p, (F) Sn 3d, (G) Cu 2p, and (H) Cu LMM. Note: open symbols, raw data; black lines, Shirley/Tougaard background; color lines, fitted peaks; red lines, enveloping curves; BE, binding energy; KE, kinetic energy; subsequent surface cleaning using argon ions was performed to remove the contaminants on the film surface before the XPS measurements.

Figure 8 shows the calibrated XPS spectra of an electrodeposited precursor film, where the subsequent surface cleaning using argon ions was done before the measurements. The full survey spectrum in Figure 8A unravels the constituent elements present in the electrodeposited precursor, which are carbon, oxygen, sulfur, copper, zinc, and tin. This observation is consistent with those of the EDX results shown in Figure 7B. The surface composition result given in Figure 8A inset was determined by quantifying the C 1s, O 1s, S 2p, Cu 3p, Sn 4d, and Zn 3d XPS lines. Compared with the bulk composition determined by EDX shown in Figure 7B, the precursor exhibits a Zn richer surface, with atomic ratios of Cu/Zn = 1.33 and Zn/Sn = 1.32, consistent with the findings presented in Section 1.2, whereas the sulfur content at the surface is very rare in comparison with that in the bulk. Also, XPS compositional analysis confirms that the precursor contains carbon and oxygen impurities, suggesting that organic tartrate and/or citrate complexes that contain both carbon and oxygen were incorporated into the precursor. The atomic ratio of carbon and oxygen C/O = 0.79, which is much larger than that of 0.66 in the tartrate (C_4_H_4_O_6_)^2−^ cluster but close to that of 0.86 in the citrate (C_6_H_5_O_7_)^3−^ cluster. As shown in Figure 8B, S 2p doublets appear at the binding energies of 161.76 eV (2p_3/2_) and 162.93 eV (2p_1/2_), where the dominant 2p_3/2_ line agrees well with most of the sulfides, such as Cu_*x*_S (161.7 eV; Larson, 1974) and SnS_*x*_ (161.7 eV; Cruz et al., 2003), indicating S^2–^ in the precursor. Besides, we do not observe any XPS peak at the higher binding energy from 172 to 167 eV coming from the oxidized sulfur (S^4+^ or S^6+^) (Hernández-Rodríguez et al., 2015, Meysing et al., 2015, Thomas et al., 1997), suggesting that no (SO_3_)^2–^, (SO_4_)^2–^, or (S_2_O_3_)^2–^ species is present in the precursor. The high-resolution C 1s spectrum in Figure 8C shows three visible peaks at the binding energies of 284.63, 286.63, and 288.76 eV, which can be assigned to C–C, C–O, and O–C=O bonds from the tartrate and citrate species, respectively (Cano et al., 2001). The O 1s spectrum in Figure 8D shows three peaks: the weak peak at 532.84 eV can be attributed to either the H_2_O or the O–C=O (C=O) bond in the organic complex clusters (Platzman et al., 2008, Zydziak et al., 2013); the dominant peak at 531.69 eV can be attributed to either the C–OH bond in the organic complex clusters or the OH^–^ in the hydroxides (Platzman et al., 2008, Šeruga et al., 1996, Zydziak et al., 2013), whereas the other intense peak at 530.19 eV can be attributed to the O^2–^ in the oxides (Cano et al., 2001, Larson, 1974, Platzman et al., 2008, Šeruga et al., 1996). These observations from the O 1s XPS peaks suggest that oxides may exist at the precursor surface, but the presence of the hydroxides and water cannot be confirmed since they produce overlapped XPS peaks with the organic complex clusters. As shown in Figure 8E, Zn 2p lines appear at the binding energies of 1,044.88 eV (2p_3/2_) and 1,021.81 eV (2p_1/2_), which agree well with the Zn^2+^ in CZTS, ZnO and ZnS (Deroubaix and Marcus, 1992), and Zn^0^ (Deroubaix and Marcus, 1992, Powell, 2012). The Auger LMM line of Zn in Figure 8F shows a peak submit at the kinetic energy of 987.75 eV, which is much smaller than that of Zn^0^ (992.4 eV; Deroubaix and Marcus, 1992, Powell, 2010), CZTS (989.46 eV, Figure S12C), and ZnS (989.4 eV; Bär et al., 2011, Deroubaix and Marcus, 1992) but close to ZnO (988.2 eV; Bär et al., 2011, Deroubaix and Marcus, 1992). At least, this indicates that Zn^2+^ rather than Zn^0^ exists in the precursor. Sn 3d_3/2_ and 3d_5/2_ lines appear at 494.73 and 486.32 eV, respectively, with a splitting energy of 8.4 eV (Figure 8F), which are close to those of the Sn^4+^ in SnO_2_ (495.17 eV/486.75 eV; Choi et al., 1996) and SnS_2_ (486.5 eV for 3d_5/2_; Cruz et al., 2003, Whittles et al., 2016) but larger than those of the Sn^2+^ in SnS (485.6 eV for 3d_5/2_; Cruz et al., 2003) and SnO (494.15 eV/485.75 eV; Choi et al., 1996) and the Sn^0^ (492.77 eV/484.36 eV; Bates et al., 1980, Wang et al., 1996). This suggests that the oxidization state of Sn in the precursor is 4+. The XPS spectrum at the Cu 2p region shown in Figure 8E demonstrates two pairs of Cu 2p lines: one pair appears at 952.37 eV for 2p_3/2_ and 932.58 eV for 2p_1/2_, with a splitting energy of 19.8 eV, indicating Cu^+^ or Cu^0^ (Chawla et al., 1992, Larson, 1974), and the other pair appears at 954.09 and 933.63 eV along with the satellite peaks that can be assigned to Cu^2+^ (Chawla et al., 1992, Larson, 1974). The Cu LMM line in Figure 8F with a peak submit at the kinetic energy of 916.57 eV suggests Cu^+^ rather than Cu^0^ (918.6 eV; Chawla et al., 1992, Deroubaix and Marcus, 1992, Larson, 1974, Wang et al., 1996); the extra shoulder peak with a kinetic energy of 918.4 eV, which does not appear in the CZTS (Figure S12E), indicates the presence of Cu^2+^ in the precursor (Chawla et al., 1992, Platzman et al., 2008, Wang et al., 1996). In short, our XPS analyses suggest that the electrodeposited precursor is possibly an inorganic-organic hybrid compound or a mixture consisting of Cu^+^, Cu^2+^, Zn^2+^, Sn^4+^, S^2–^, O^2–^, (C_6_H_5_O_7_)^3−^, and (C_4_H_4_O_6_)^2−^ species.

### Device Fabrication and Characterization

**Figure 9:**
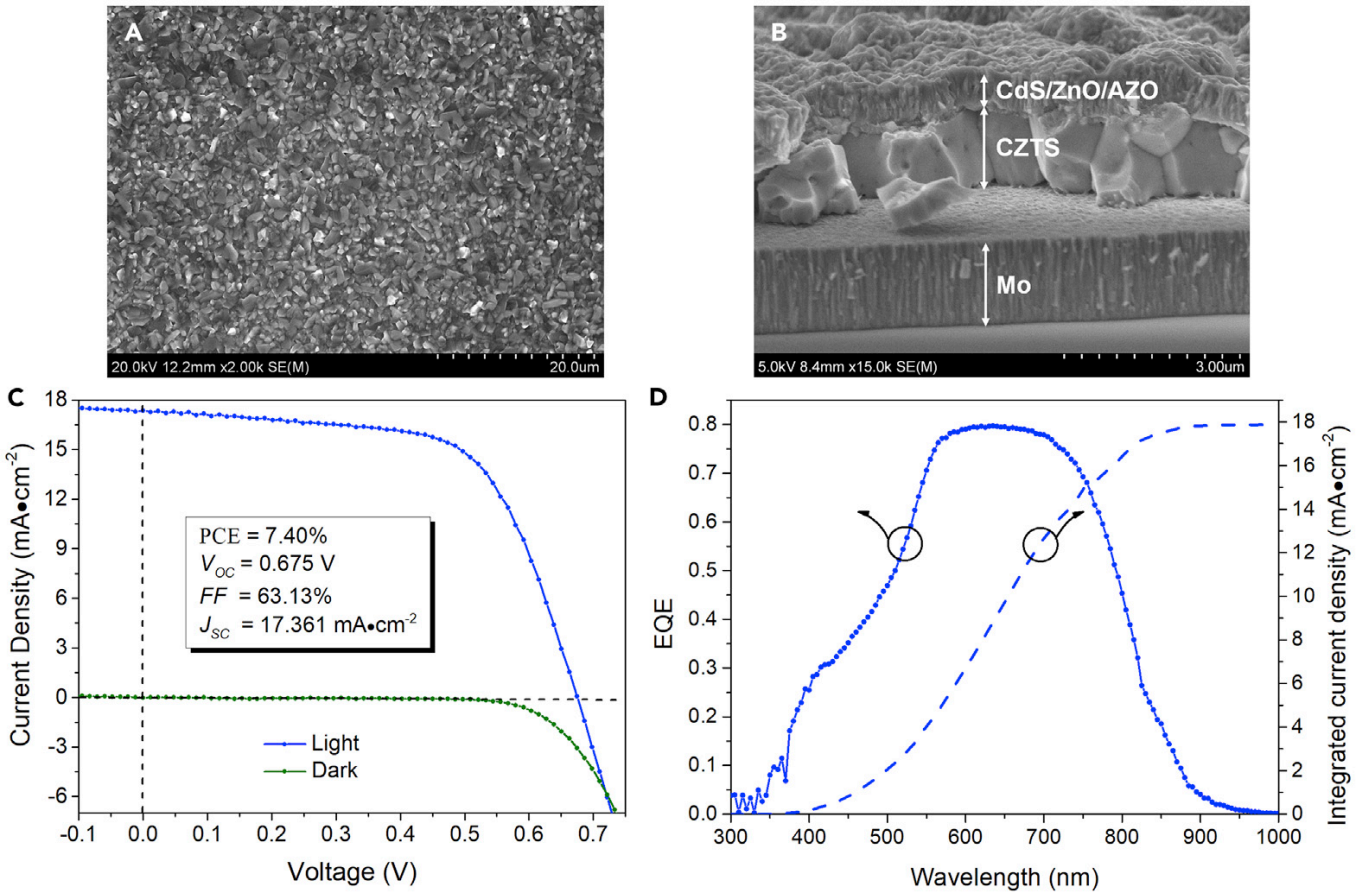
SEM Characterization and Device Characteristic of Cu_2_ZnSnS_4_ (CZTS) Absorber Film and Solar Cell based on Mo Substrate (A) Top-view morphological SEM image of our sulfurized CZTS film; (B) cross-sectional SEM image of a finished glass/Mo/CZTS/CdS/ZnO/AZO solar cell; (C) current-voltage (J–V) characteristic under AM 1.5 global illumination and in dark and (D) spectral response of external quantum efficiency (EQE) as well as EQE integrated photocurrent density of our record glass/Mo/CZTS/CdS/ZnO/AZO solar cell. Note: CZTS film was annealed using sulfur powder in a mixed nitrogen (95%) and hydrogen (5%) environment.

The electrodeposited precursors based on Mo, ITO, and FTO substrates were then sulfurized at high temperatures for the crystallization of the kesterite CZTS phase. Figure 9A shows the morphological SEM image of a CZTS film on the Mo substrate annealed in the environment containing a handful of tin sulfide vapor, sufficient sulfur vapor, as well as 5% hydrogen. It can be seen that the sulfurized film is compact and free from pinholes and secondary phases atop the film, with grain sizes varying from 0.6 to 2 μm. Raman spectrum given in Figure S13 further confirms that our CZTS film surface is made of pure kesterite phase, which is necessary to construct a high-quality p-n junction for a solar device. As shown in Figure 9B, the large CZTS grains extend through the entire absorber layer thickness, unlike the fine-grained bottom layer issue rife in the films sulfurized using sulfur vapor alone (Figure S14A; Ge et al., 2014b, Ge et al., 2016a). This suggests that the introduction of hydrogen into the annealing environment can promote element diffusion and remove the foreign carbon and oxygen impurities in the precursor. The XPS spectra of the sulfurized film (Figures S12F and S12G) further confirm that there is little trace of carbon and oxygen remaining in CZTS. Using this CZTS absorber, we fabricated solar cells with a device structure of Mo/CZTS/CdS/ZnO/AZO (Al-doped ZnO), where a CdS buffer layer was deposited by the chemical bath method and resistive and conductive ZnO window layers were deposited by sputtering. Before the deposition of ZnO overlayers, we annealed the CZTS/CdS junction at 150°C for 30 min in a vacuum furnace to remove the chemical impurity in the chemical-bath-deposited CdS and to enhance the junction quality (Ge and Yan, 2017). The best mini device with an area of 0.08 cm^2^ delivers a power conversion efficiency (PCE) of 7.4%, an open circuit voltage (V_OC_) of 0.675 V, a fill factor (FF) of 63.13%, and a short circuit current density (J_SC_) of 17.361 mA⋅cm^−2^ under the simulated AM 1.5 illumination (Figure 9C). Figure 9D shows the external quantum efficiency (EQE) data of this record cell. The EQE curve exhibits no waning trend after 550 nm; on the contrary, it shows a plateau in the spectral region from 550 to 700 nm with an EQE maximum of ∼0.8. The inclined EQE curve usually reflects a small photocarrier diffusion length making the built-in field difficult to collect the photocurrent generated at the rear side of the absorber. The observed plateau in our EQE curve suggests that our synthesized CZTS absorber is of premium electronic quality for photovoltaics. The EQE curve exhibits a rapid fall after 750 nm, indicating the absorption edge of CZTS. A 1.49 eV bandgap was estimated for CZTS from the first derivative of the EQE data. The short circuit current density integrated from the EQE data, 17.87 mA⋅cm^−2^, is consistent with the current-voltage measurement in Figure 9C. In addition, the mini cells from the same large piece sample indeed show considerable fluctuations in their PCEs, varying from 5.7% to 7.4%, with their V_OC_s varying from 0.70 to 0.63 V. We further find that a small grain bottom layer with a thickness of 200–250 nm may sometimes appear at the absorber rear side in these ∼6% efficient mini cells, atop which the large grain layer has a relatively larger layer roughness (Figure S15). We cannot exclude the likelihood of the segregation of spurious phases, e.g., ZnS, in the small grain layer, which cannot be detected by Raman spectra from the film surface side. Thus, these may inevitably cause the degraded device parameters of these mini cells, in particular FF, consequently leading to relatively lower PCEs.

Table S2 presents the device parameters of some widely recognized CZTS solar cells in the literature for comparison with our best electrodeposited solar cell. Indeed, our best electrodeposited solar cell demonstrates its device parameters, in terms of V_OC_ and FF, comparable with the high-performance CZTS solar cells that were prepared by the vacuum-based methods. Our J_SC_ is 2–3 mA⋅cm^−2^ smaller than that of the high-performance CZTS cells with the antireflection coatings (ARCs; Table S2), and as a result, our PCE is ∼2% smaller. Actually, our EQE maximum, as high as 80%, has reached the limit for a CZTS thin-film solar device without the ARC coating. This suggests that our CZTS absorber prepared using alloy ED plus post-sulfurization annealing in a hydrogen-containing environment demonstrates the photovoltaic characteristic as superb as the counterparts obtained by vacuum-based methods. Higher J_SC_ and better PCE are anticipated for our electrodeposited solar cell if ARC coatings could be employed in the future.

Our electrodeposited precursors based on ITO and FTO substrates were annealed in sulfur vapor without hydrogen. Hydrogen was not used in the annealing because it can ruin the transparent conductive oxide-based back contacts at high temperatures (Ge et al., 2014a, Ge et al., 2015b, Ge et al., 2015a). Figures S14B and S14C show that fine-grained bottom layers are rife in these sulfurized CZTS absorbers based on ITO and FTO substrates. The chemical components of our electrodeposited precursors may directly result in this fine-grained bottom layer issue, because the chemical impurities at the film rear side cannot be exposed to sulfur vapor substantially and be completely removed. Nonetheless, the finished CZTS solar cells with ITO and FTO back contacts still deliver power conversion efficiencies exceeding 5% under the simulated AM 1.5 illumination from the AZO front side (Figure S16A). Since these cells are based on transparent back electrodes, the illumination can also be from the ITO or FTO rear side. Under rear illumination, the solar devices can still deliver some photovoltages and photocurrents as shown in Figures S16B and S16C. This device structure using the transparent conductive oxides as both front and back contacts may function as bifacial, semitransparent, or tandem sub-cell applications.

### Conclusions

This treatise aims at developing the multinary alloy electrodeposition (ED) technology for the low-cost industrial manufacture of thin-film solar cells. Choosing kesterite Cu_2_ZnSnS_4_ as a case study, we have systematically investigated complicated deposition conditions in detail, such as bath composition, bath agitation, plating time, distance between the working and counter electrodes, additives, and bath stability, and solved the technical difficulties all the while perplexing alloy ED in the practical aspects on how to control the alloy composition and the deposit morphology simultaneously. A series of refined plating parameters and an optimized electrolyte recipe have been formulated, achieving electrodeposits with superb layer uniformity in film appearances and compositions, which is able to rival the precursors deposited by vacuum-based methods. The acquired experiences and understandings and new discoveries regarding the co-plating process shine light on the development of new co-plating systems for other multi-component thin-film solar cells, in particular for those made of the elements with large ED potential differences, such as conventional copper-indium-gallium selenide and state-of-the-art copper-strontium-tin chalcogenides (Ge et al., 2016b, Ge et al., 2016c, Ge et al., 2017a, Ge et al., 2017b).

Specifically, we found that the deposit composition can be tuned by mass transport control, wherein the Zn ion with a much negatively deposition potential may preferentially deposit because of its very high bath concentration and only when the deposition of Cu and Sn ions arrived at their diffusion-limited rates. Under these circumstances, both prolonging the plating time and minimizing the working-counter electrode distance (amounting to increasing the current density) can increase the Zn content in the electrodeposits. Besides, for the first time, it has been found that a handful of sodium thiosulfate additive (5 mM) can act as a brightener, a leveling agent, and a hydrogen inhibitor, leading to uniform, smooth, compact, and nanocrystalline (grain size <40 nm) electrodeposits. Meanwhile, we also for the first time found that a little bit of sodium sulfite additive (1 mM) can function as an effective stabilizer, leading to a satisfactory stability up to 1 day of the mixed thiosulfate-sulfite electrolyte bath.

We also found that the electrodeposited precursor contains foreign impurity elements of carbon and oxygen, which primarily come from the citrate and/or tartrate complexing agents. Post-annealing in the environment containing both hydrogen and sulfur can effectively remove these impurity elements and yield a uniform and compact absorber layer with large Cu_2_ZnSnS_4_ grains (average size ∼1 μm) extending through the entire absorber layer thickness. Solar devices with the configuration Mo/Cu_2_ZnSnS_4_/CdS/ZnO/Al-doped ZnO have been fabricated, delivering a 7.4% power conversion efficiency.

## Methods

All methods can be found in the accompanying Transparent Methods supplemental file.

## Supplemental Information

Document S1. Transparent Methods, Figures S1–S16, and Tables S1 and S2
