# Supplementary material for: Controllable Multinary Alloy Electrodeposition for Thin-Film Solar Cell Fabrication: A Case Study of Kesterite Cu2ZnSnS4
Source: iScience. 2018 Mar 8;1:55–71. doi: 10.1016/j.isci.2018.02.002 (PMC6135938; doi:10.1016/j.isci.2018.02.002)
Supplement: Document S1. Transparent Methods, Figures S1–S16, and Tables S1 and S2 [file mmc1.pdf]

ISCI, Volume *1*

Supplemental Information

Controllable Multinary Alloy Electrodeposition for Thin-Film Solar Cell Fabrication: A Case Study of Kesterite Cu_2ZnSnS_4

Jie Ge and Yanfa Yan

Transparent Methods

Chemical reagents and make–up of electrolyte for device–grade precursor electrodeposition

Copper (II) sulfate pentahydrate (Acros Organics >98%), zinc sulfate heptahydrate (Acros Organics >99%), tin (II) sulfate (Acros Organics >97%), trisodium citrate dihydrate (Sigma–Aldrich >99%), Dipotassium tartrate hemihydrate (Sigma–Aldrich >99%), sodium thiosulfate pentahydrate (Sigma–Aldrich >99.5%) and sodium sulfite (Sigma–Aldrich >98%) were used as obtained. The dissolution of tin sulfate needs the presence of citrate and tartrate in the aqueous solution. Thus, trisodium citrate (110 mM) and dipotassium tartrate (16 mM) were dissolved by 1 L of highly purified water (resistance 18 MΩ) at the very beginning. Then, tin sulfate (6 mM) and zinc sulfate (50 mM) and copper sulfate (7 mM) were sequentially added into the bath until the previous chemical was fully dissolved. Sodium thiosulfate (5 mM) was added into the bath after the dissolution of metal salts and agitation was kept for about 15 min until the bath color completely changed from sky–blue to greenly blue. Sodium sulfite (1 mM) was added and dissolved at last. All the operations for the electrolyte make–up require strong agitation.

Electrodeposition for device–grade precursors

The substrates for precursor electrodeposition were based on the substrates of the DC–sputtered Mo glass and the commercial indium–tin oxide coated glass (ITO, NSG, 8–10 Ω per square) and the fluorine–doped tin oxide coated glass (FTO, NSG, TEC7). Electrodeposition of precursor films was carried out using a potentiostat (ModuLab XM ECS Electrochemical Test System, Ametek Scientific Instruments) and a conventional three–electrode assembly with a Ag/AgCl (1 M KCl aqueous solution) reference, an inert Pt–coated Ti plate (15×12 cm^2) counter electrode, and a working electrode Mo or ITO or FTO coated glass substrate. The working and counter electrodes were vertically loaded into the bath with a 4 cm equidistance from each other. The precursor film on Mo or ITO or FTO substrate was deposited at –1.135 or –1.145 or –1.16 volts versus Ag/AgCl in potentiostatic mode for 30 min at room temperature without agitation. Prior to the electrodeposition of the precursor film, the fresh electrolyte was passed through by a low current for necessary timings, which is referred to as pre–electrolysis (or dummying).

Heat treatment and device fabrication

The sulfurization was carried out in a tube furnace at 540 (580) °C for 30 (15) min to form the kesterite Cu_2ZnSnS_4 (CZTS) phase. The precursor samples were loaded into a graphite box together with 0.5 g sulfur powder and 20 mg SnS power. For the crystallization of the CZTS film on Mo substrate, a 50 sccm mixed gas of nitrogen (vol. 95%) and hydrogen (vol. 5%) was flowed into the tube furnace as the carrier gas during the annealing. For the crystallization of CZTS films on ITO and FTO substrates, a 50 sccm pure argon was flowed into the tube furnace as the carrier gas during the annealing. The gas pressure in the annealing chamber was maintained at about 500 Torr. The *n–type* CdS layer (~50 nm) was deposited on the just–sulfurized CZTS absorbers to create the *p–n* junction. The growth of CdS uses chemical solutions of cadmium acetate (0.024 mM), thiourea (15.5 mM), ammonium acetate (21 mM), and ammonium hydroxide (1 M) at 68 °C. The CdS/CZTS junction was then annealed at 150 °C in nitrogen for 30 min to remove the impurity in the chemical solution deposited CdS layer and improve the junction quality. Window layers of resistive ZnO and conductive aluminum–doped ZnO (AZO) were sequentially sputtered at ambient temperature through an aperture mask. No metal grid and antireflection coating was deposited on each individual cell.

Characterization

The linear sweep voltammetry scans of the electrolyte baths were carried out using a Pt wire as the counter electrode, Mo coated glass slices as the working electrodes, and a Ag/AgCl reference electrode (scan rate 10 $mV \cdot s^{-1}$). Compositional results were acquired by energy dispersive X–ray spectroscopy (EDX) equipped with a field–emission scanning electron microscope (FE–SEM) for the precursor. Cross–sectional and surface morphological SEM images were conducted using the Hitachi S4800 FE–SEM. X–ray diffraction (XRD) data of

the precursors were collected using a Rigaku Ultima III diffractometer with Cu Kα lines (0.15418 nm) in θ–2θ scans operated at 40 kV and 44 mA. Phase identification of XRD data is referenced to $In_{1.88}Sn_{0.12}O_3$ (PDF 97−005−0849) for ITO substrate, SnO_2 (PDF 97–026–2768) for FTO substrate, and Mo (00–042–1120) for Mo substrate using a Jade 2010 software. X−ray photoelectron spectroscopy (XPS) was conducted using a PHI Quantum 2000 spectrometer fitted with the monochromatic Al Kα X−rays (1486.6 eV). Ar^+ beam (200 eV) was used to remove the contaminants (~10 nm) on the precursor surface prior to the XPS measurement. The surface composition of the electrodeposited precursor film was determined by quantifying the Cu 3p, Sn 4d, and Zn 3d XPS lines because the respective photoelectrons have high and very similar kinetic energies. Thus, the corresponding peak intensities are less influenced by surface contaminations than any other set of XPS lines, and transmission and mean free path variations can be neglected.[1] The intensities of XPS lines were fitted with Voigt line profiles and Shirley/Tougaard backgrounds by the software of CasaXPS. The high resolution XPS spectra of element Cu, Zn, Sn, S, and O were fitted using a mixed Gaussian–Lorentzian GL(30) lines, while the VS(,,,,)SGL(14) functions were used to fit the asymmetric line–shapes of the C 1s peaks. Confocal Raman spectroscopy was carried out using a 632.8 nm laser (HORIBA Scientific), with the Raman shift calibrated by a Si sample at 520.4 cm^{-1}. Film thickness was determined by a Dektat 8 Stylus Profiler. Current–voltage characteristics (J−V) of the solar devices were measured under simulated AM 1.5 global spectrum with the irradiance set to 100 $mW\cdot cm^{-2}$ (PV Meas. Inc.). External quantum efficiency (EQE) measurements were performed by a single source illumination system combined with a monochromator (PV Meas. Inc.).

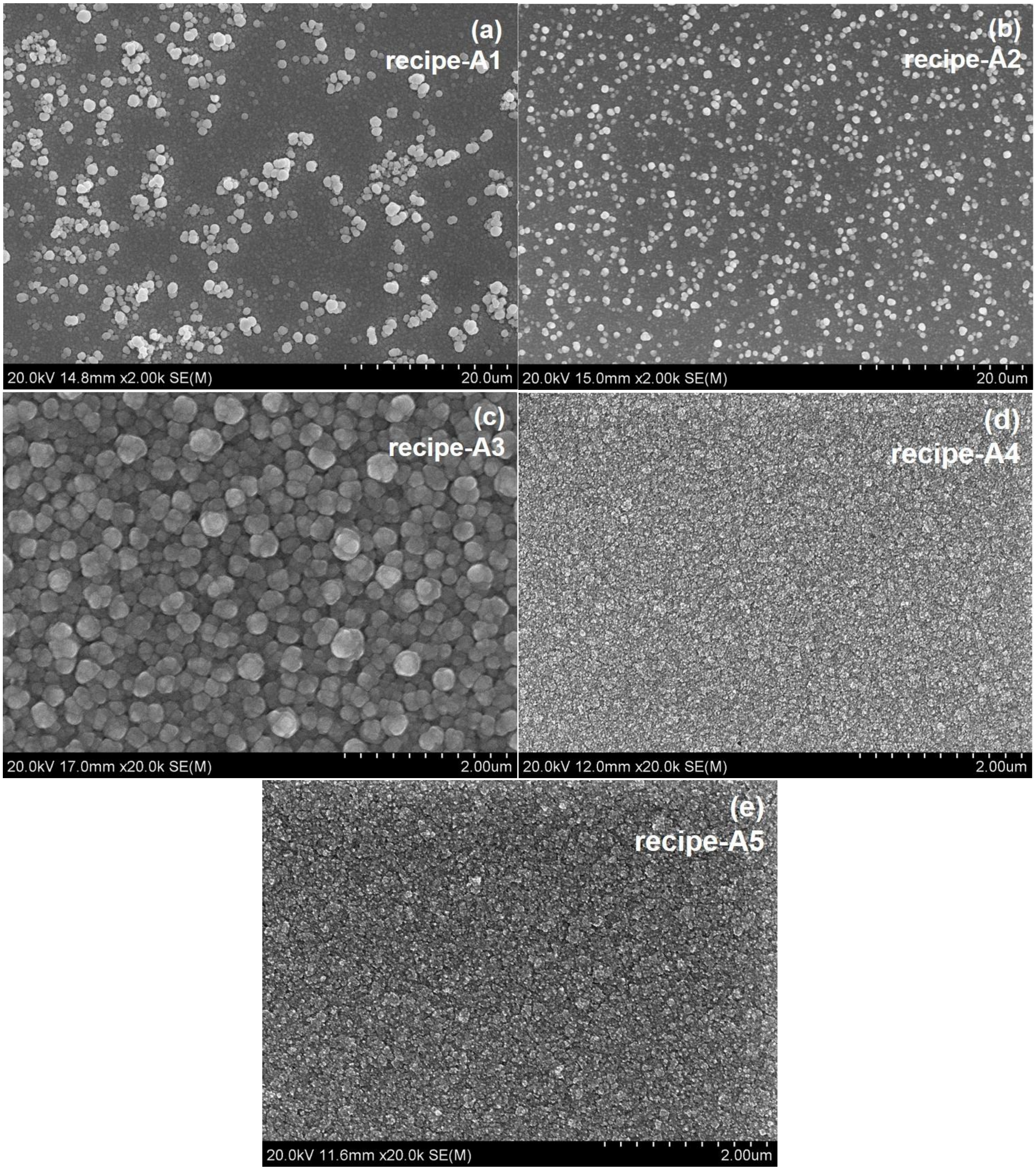

Figure S1. Top–view SEM images of the electrodeposits using the electrolyte baths (1 L in volume) based on various chemical recipes, Related to **Table 1**

(a) recipe–A1; (b) recipe–A2; (c) recipe–A3; (d) recipe–A4; (e) recipe–A5.

Note: the other plating parameters of the electrodeposits include (1) without agitation or bath heating, (2) 30 min plating time, and (3) 4 cm working–counter electrode distance.

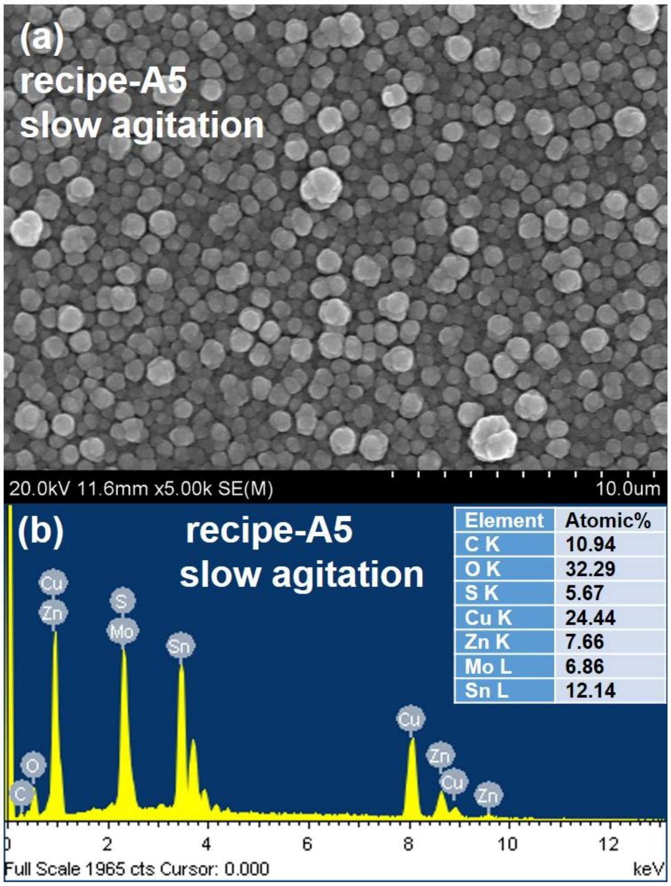

Figure S2. Top–view SEM image (a) and EDX spectrum & composition result (b) of an electrodeposit obtained from chemical recipe–A5 electrolyte with the bath (1 L in volume) being slowly stirred during the deposition, Related to **Table 1**

Note: the other plating parameters of electrodeposits include 30 min plating time and 4 cm working–counter electrode distance.

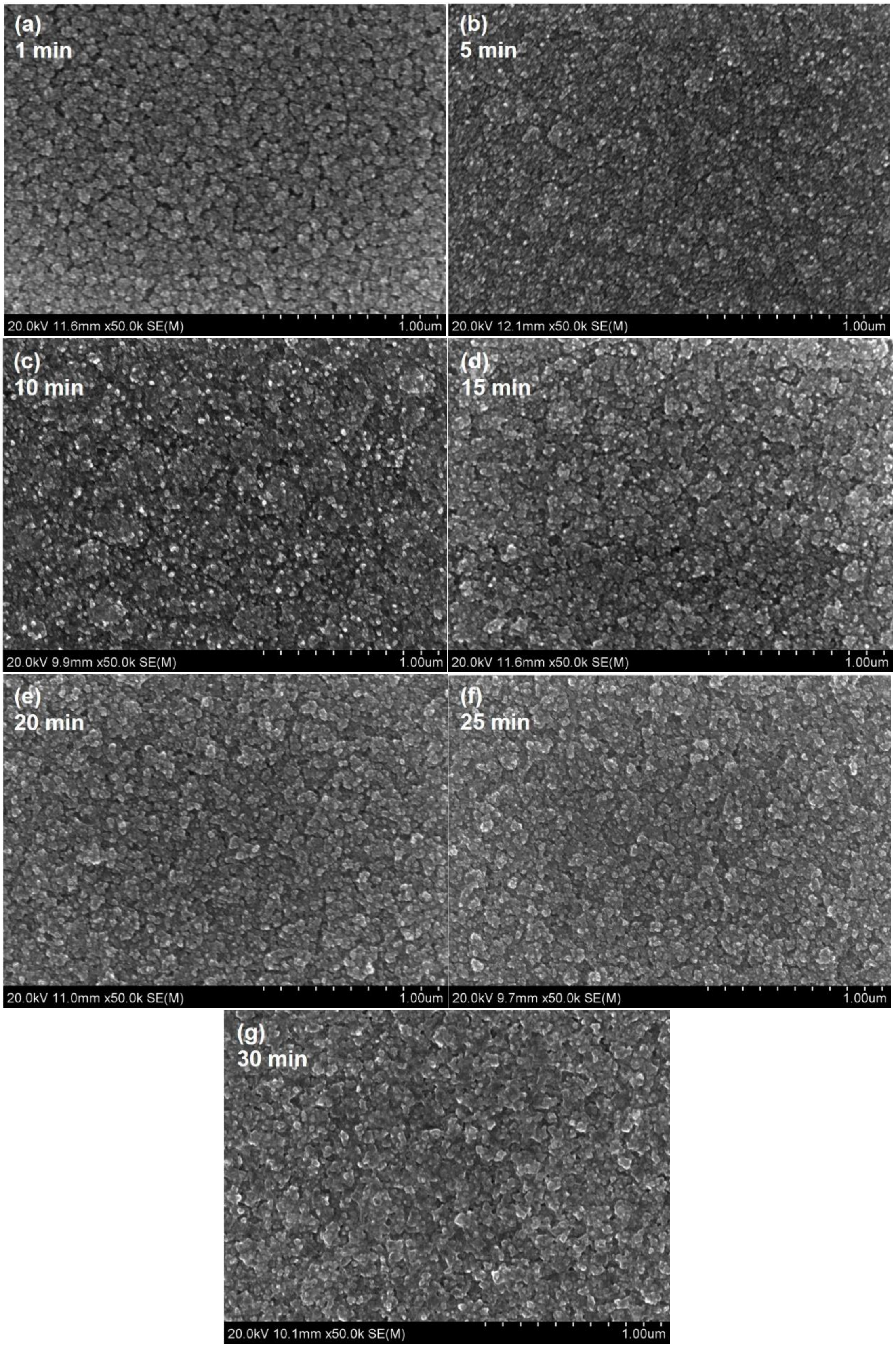

Figure S3. Top–view SEM images of the electrodeposits using various plating time, Related to **Figure 1**

(a) 1 min; (b) 5 min; (c) 10 min; (d) 15 min; (e) 20 min; (f) 25 min; (g) 30 min.

Note: electrolyte bath (1 L in volume) based on recipe–A5; the other plating parameters include (1) without agitation or bath heating and (2) 4 cm working–counter electrode distance.

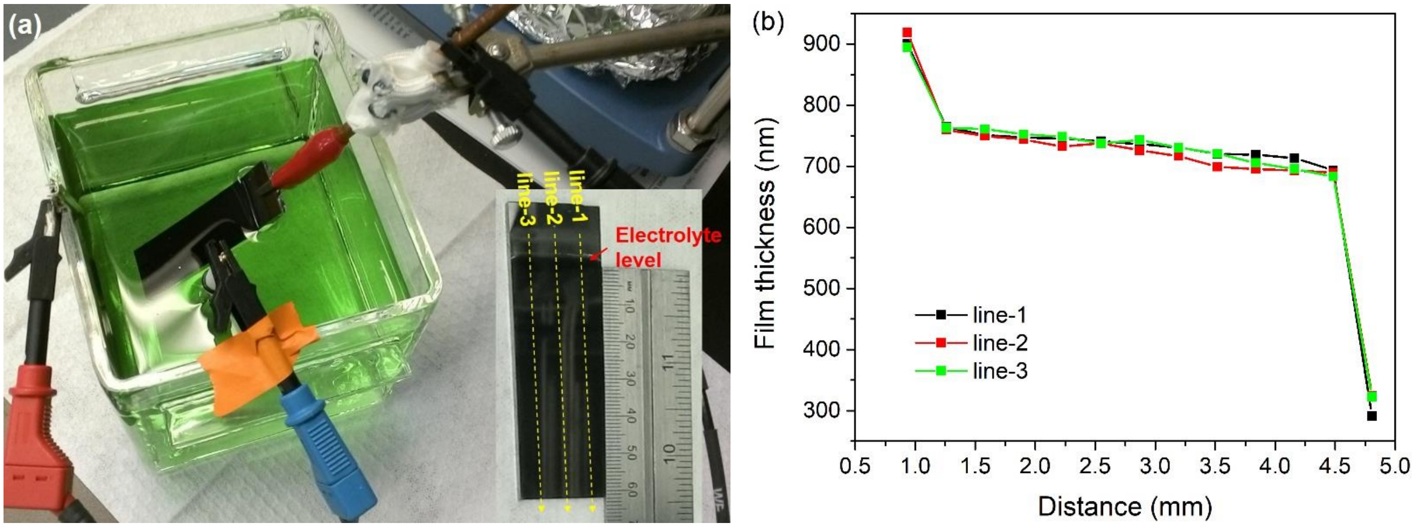

Figure S4. Photos of experimental set–up of Hull cell and an electrodeposit on a Mo substrate (1 by 3 inches) obtained from the Hull cell configuration (a); the film thickness variance along the length of the deposit [see line-1, line-2, and line-3 in panel (a) inset] showing the relation with the working-counter electrode distance (b), Related to **Figure 2**.

Note: electrolyte bath (1 L in volume) based on recipe–A5; the other plating parameters includes (1) without agitation or bath heating and (2) 30 min plating time.

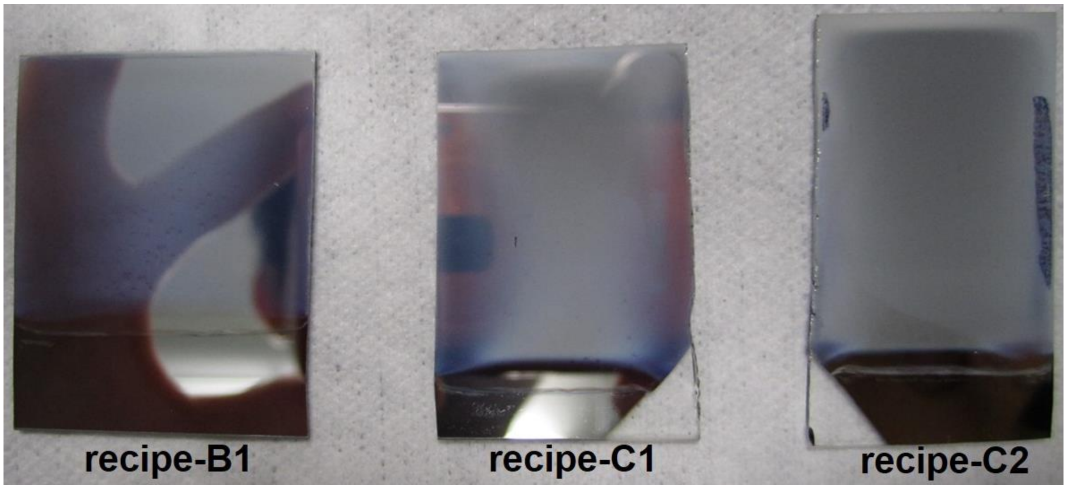

Figure S5. Photos of the electrodeposits from chemical baths (1 L) of recipe–B1, C1 and C2, Related to **Table 2** and **Figure 3**.

Note: all the deposits exhibit nearly stoichiometric metal compositions that are required for kesterite absorbers; the other plating parameters include (1) without agitation or bath heating, (2) 30 min plating time, and (3) 4 cm working–counter electrode distance.

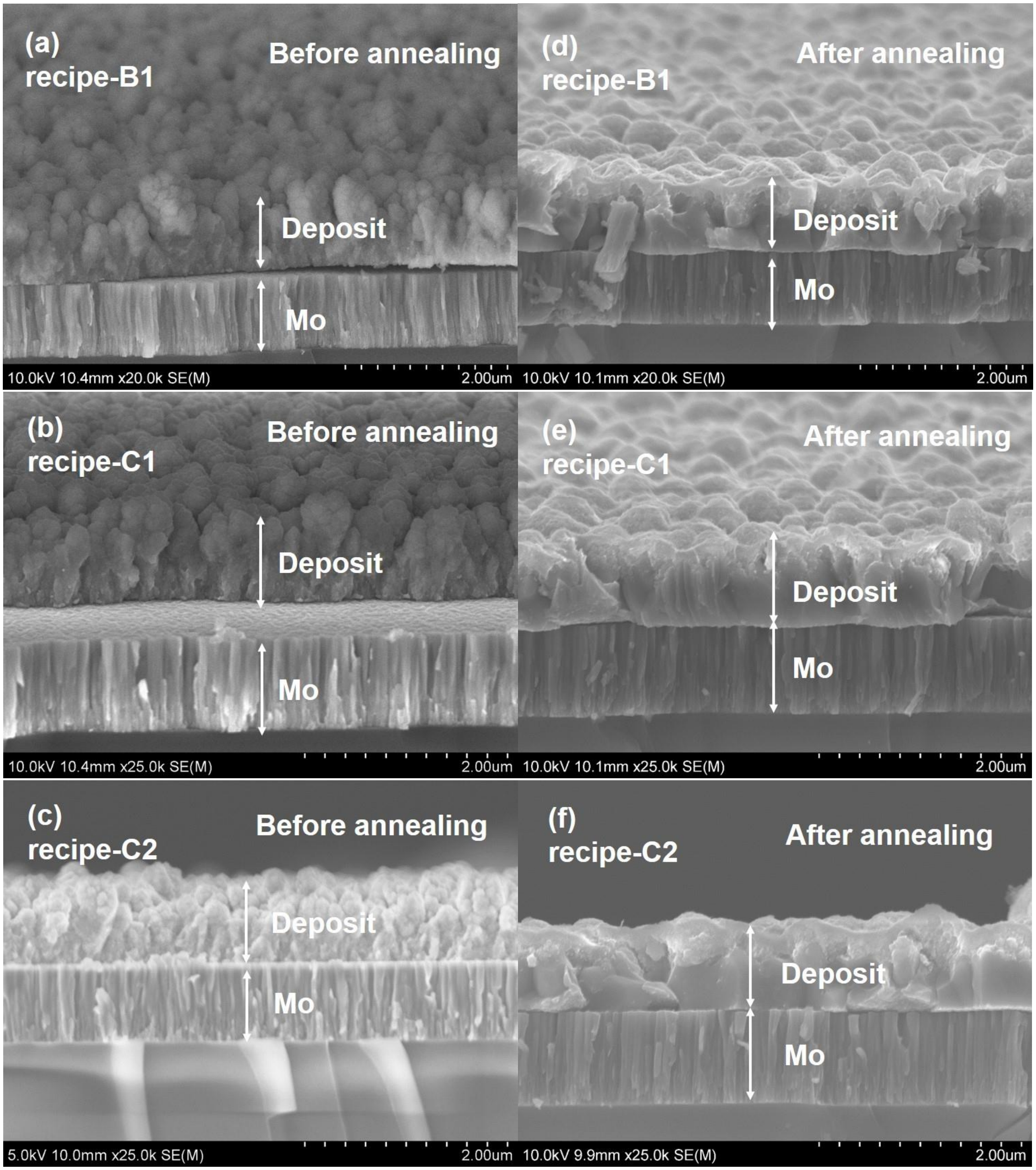

Figure S6. Cross–sectional SEM images of the electrodeposits from the different electrolyte baths without sodium sulfate additive, Related to **Table 2** and **Figure 3**.

The fresh electrodeposits without pre–annealing obtained from recipe–B1 (a), recipe–C1 (b), and recipe–C2 (c). The corresponding counterparts after the annealing at 320 °C for 30 min in pure argon environment (d), (e), and (f).

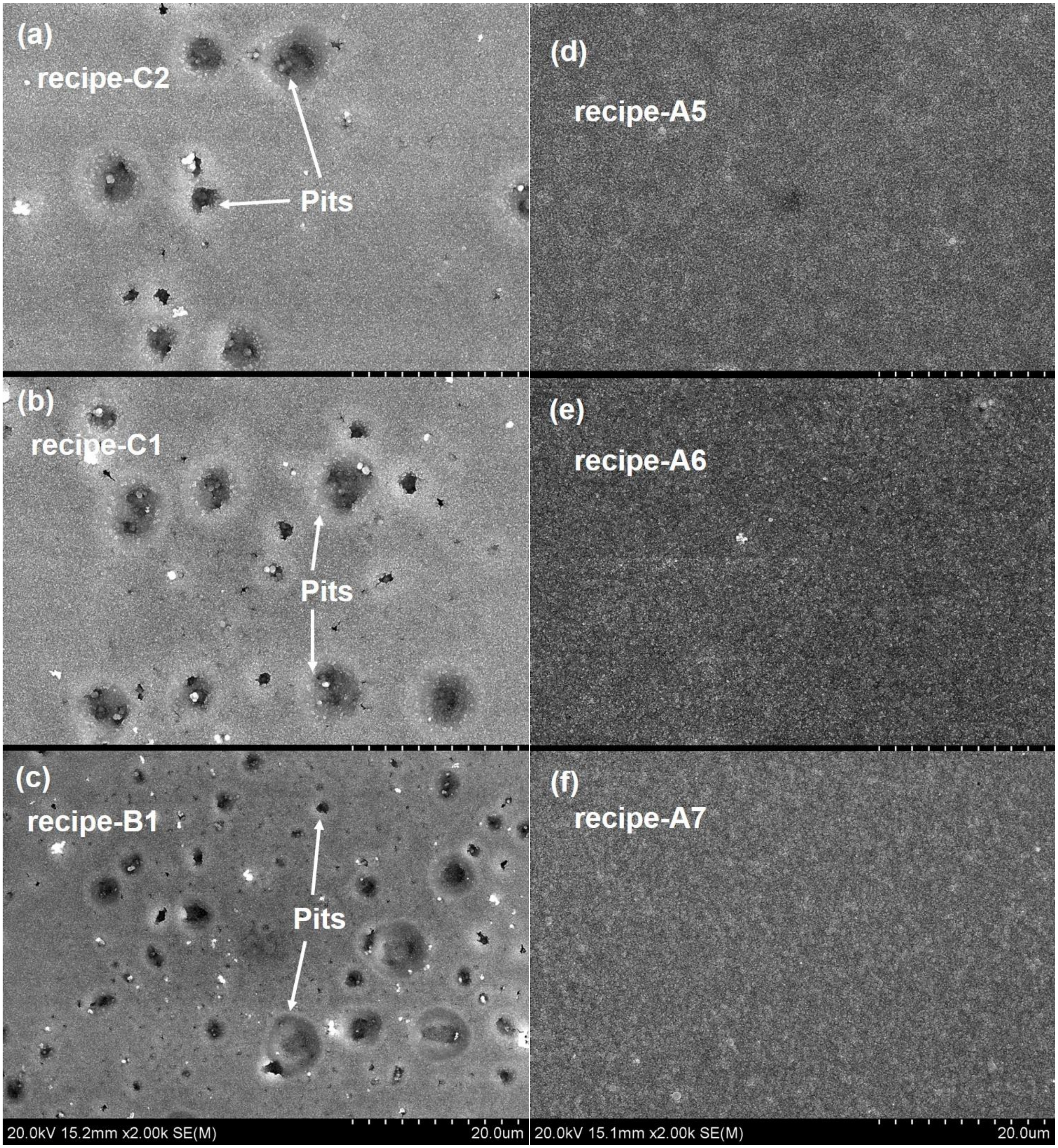

Figure S7. Top–view SEM images at low magnifications of the electrodeposits from the chemical baths without sodium thiosulfate additive of recipe–C2 (a), C1 (b), and B1 (c) and the electrodeposits from the baths containing 5 mM thiosulfate additive of recipe–A5 (d), A6 (e), and A7 (f), Related to **Figure 4**.

Note: the SEM images of panels (a), (b), and (c) correspond to the surface areas where apparent hydrogen evolution occurred during the electrodeposition.

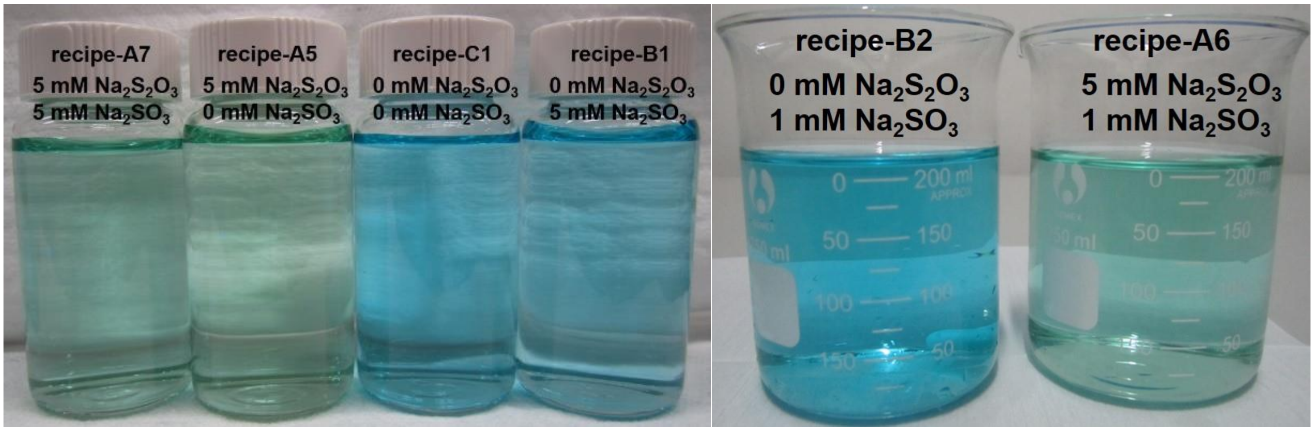

Figure S8. Photos of the fresh electrolyte solutions based on different chemical recipes with and without sodium thiosulfate and sodium sulfite additives, showing that the addition of sodium thiosulfate additive can change the bath color from sky–blue to greenly blue, Related to **Table 2** and **Figure 5**

Note: 1. these photos were taken 15 min after the make–ups of electrolyte solutions; 2. the original electrolyte solutions are all 1 L in volume, which were prepared in a large rectangular container (see Figure S4); then, small portions of these just–made electrolyte solutions were transferred into 20 ml bottles (left photo) and 300 ml beakers (right photo).

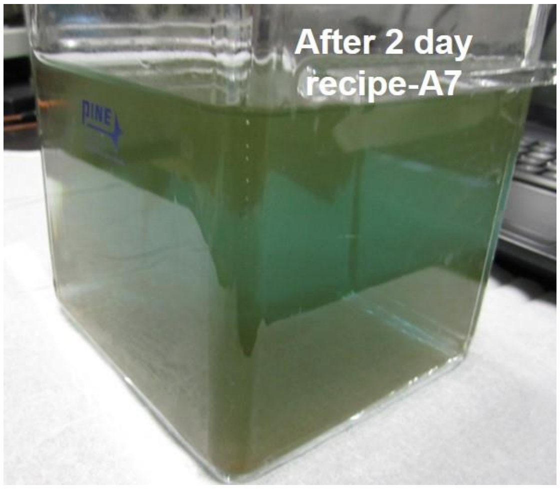

Figure S9. Photo of a degraded electrolyte bath (1 L) based on recipe–A7 after 2 days, Related to **Table 2** and **Figure 5**

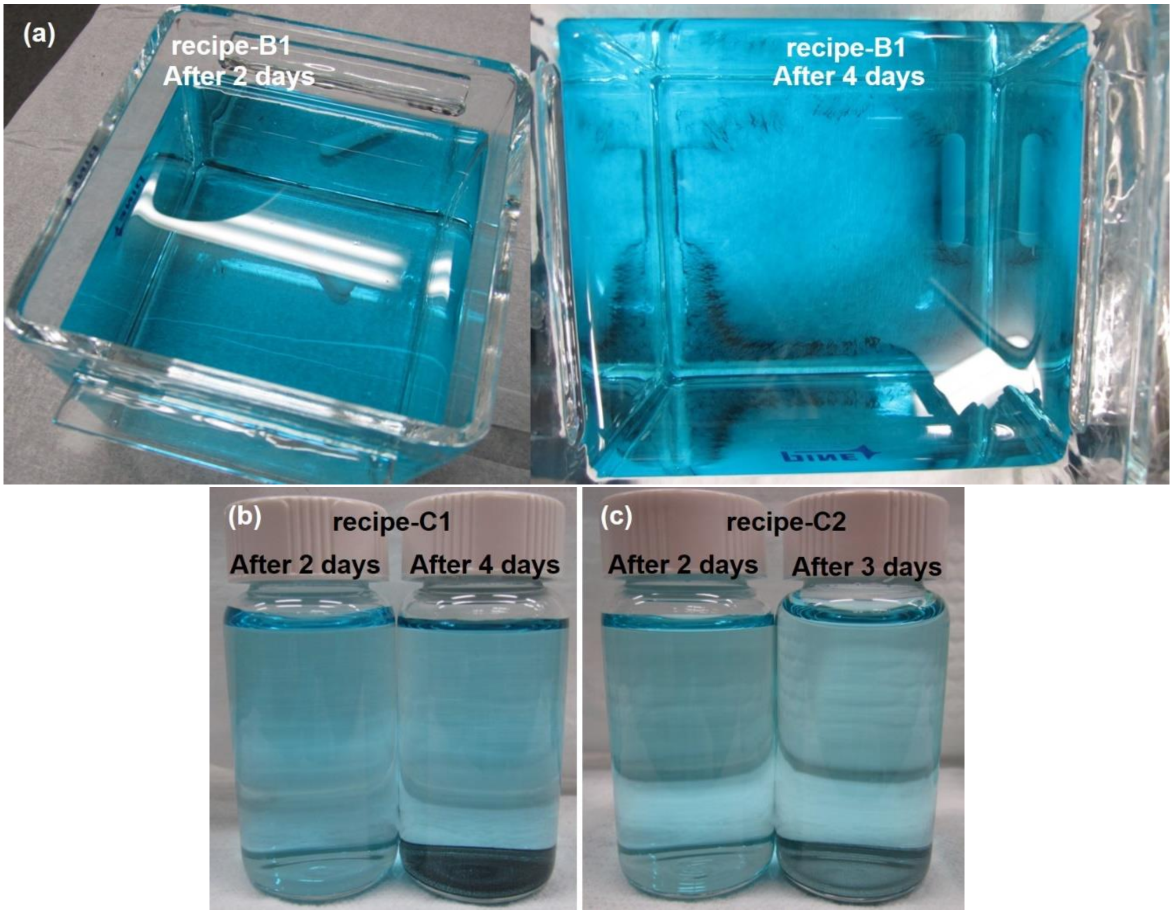

Figure S10. Photos of the electrolyte solutions based on different chemical recipes without sodium thiosulfate additive after different storage durations, showing the bath stability, Related to **Table 2** and **Figure 5**

(a) Bath of recipe–B1 after 2 days and 4 days.
(b) Bath of recipe–C1 after 2 days and 4 days.
(c) Bath of recipe–C2 after 2 days and 3 days.

Note: 1. the original electrolyte solutions are all 1 L in volume, which were prepared in a large rectangular container as shown in panel (a); 2. small portions of electrolyte solutions of recipe–C1 and C2 were transferred into 20 ml bottles [panels (b) and (c)] after they were just made.

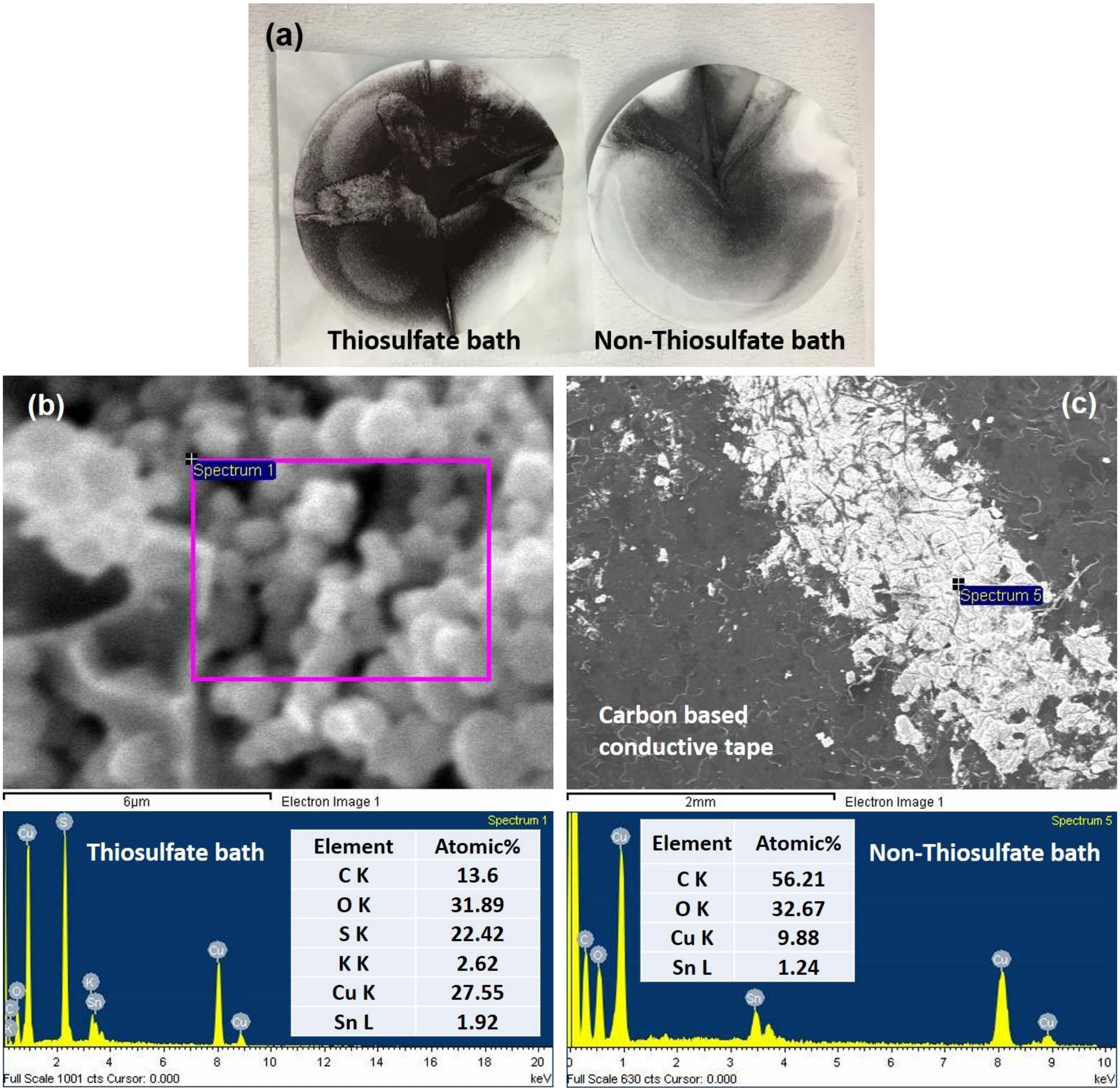

Figure S11. Digital photo showing the filtered and washed precipitates generated from the electrolyte baths with and without thiosulfate additive after 3 days storage (a), SEM images and EDX spectra showing the compositions of the precipitates (b) and (c), Related to **Figure 5**.

Table S1. Atomic ratios of the metal elements within a 3 by 2.5 cm^2 region of an electroplated kesterite precursor on a 2 by 2 squared inches Mo substrate, Related to **Figure 6**

Cu/Zn	Zn/Sn	Cu/Sn
1.683	1.03	1.73
1.676	1.042	1.75
1.68	1.031	1.73
1.687	1.035	1.7
1.70	1.04	1.77
1.698	1.052	1.79
1.692	1.046	1.77
1.688	1.035	1.75
1.693	1.038	1.76
1.683	1.041	1.75
1.69	1.05	1.775
1.692	1.051	1.778
1.687	1.047	1.766
1.689	1.04	1.757
1.685	1.039	1.751
1.68	1.05	1.764
1.66	1.069	1.775
1.677	1.063	1.783
1.685	1.059	1.784
1.69	1.063	1.796
1.68	1.07	1.798
1.7	1.06	1.802
1.67	1.04	1.74
1.69	1.045	1.766
1.71	1.05	1.80
1.705	1.06	1.81
1.69	1.055	1.783
1.685	1.053	1.774
1.672	1.065	1.781
1.68	1.07	1.798
1.667	1.063	1.772
1.676	1.06	1.776
1.685	1.057	1.781
1.69	1.051	1.776
1.688	1.05	1.772
1.681	1.054	1.772
1.679	1.06	1.78
1.68	1.067	1.79
1.675	1.055	1.77
1.683	1.06	1.78
1.71	1.03	1.76
1.7	1.06	1.80

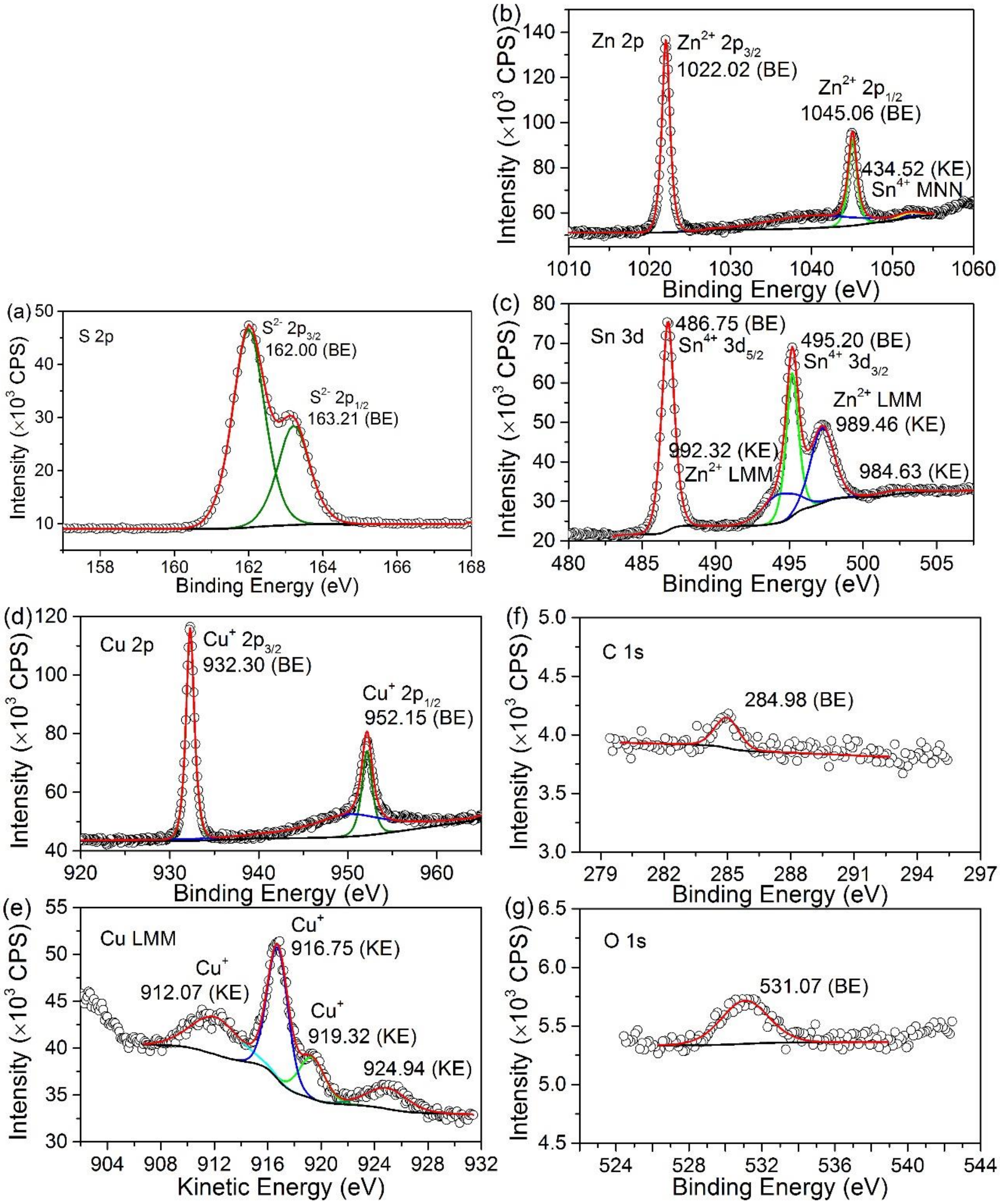

Figure S12. X–ray photoelectron spectroscopic (XPS) measurements of a sulfurized CZTS film: detailed measurements at the region of S 2p (a), Zn 2p (b), Sn 3d (c), Cu 2p (d), Cu LMM (e), C 1s (f), and O 1s (g), Related to **Figure 8**

Note: open symbols−raw data; black lines−Shirley/Tougaard background; colorful lines−fitted peaks; red lines−enveloping curves; BE–binding energy; KE–kinetic energy; subsequent surface cleaning using argon ions was performed to remove the contaminants on the film surface prior to the XPS measurements.

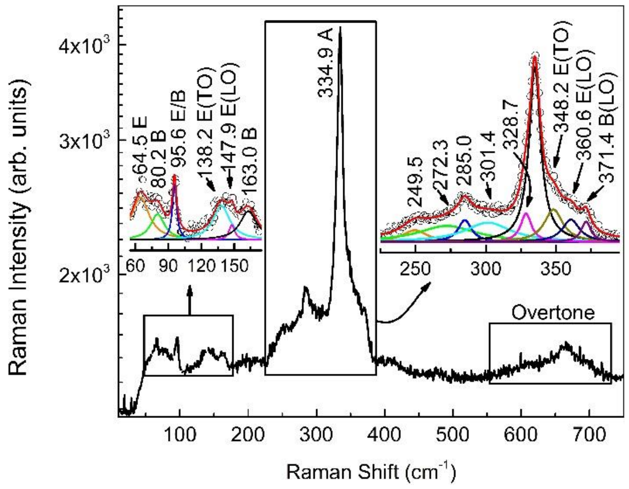

Figure S13. Raman spectrum of a sulfurized CZTS film, Related to **Figure 9**

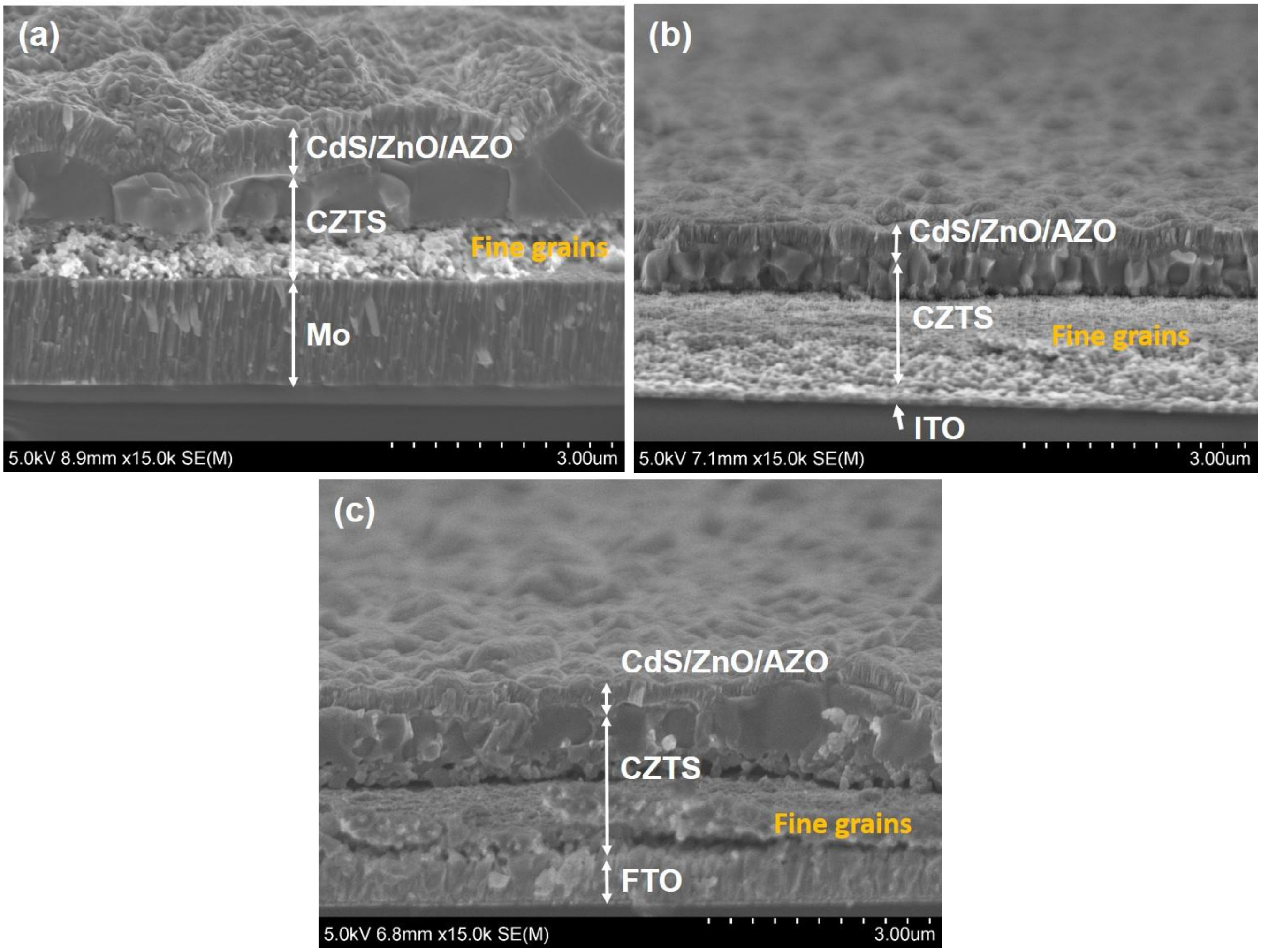

Figure S14. Cross–sectional SEM images of a finished glass/Mo/CZTS/CdS/ZnO/AZO solar cell (a), glass/ITO/CZTS/CdS/ZnO/AZO solar cell (b), and a glass/FTO/CZTS/CdS/ZnO/AZO solar cell, Related to **Figure 9**

Note: all the CZTS absorber films were sulfurized in the pure argon environment without hydrogen.

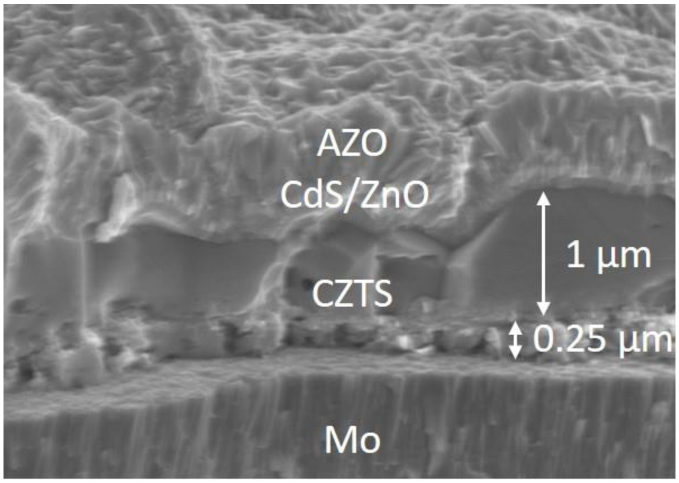

Figure S15. Cross–sectional SEM image showing the presence of a small grain bottom layer at the rear side of the CZTS absorber sulfurized in the hydrogen–containing environment, Related to **Figure 9**

Table S2. Comparison of device parameters of selected widely-recognized Mo/Cu_2ZnSnS_4/CdS/ZnO thin–film solar cells in the literatures,[a] Related to **Figure 9**

PCE (%)	V_{OC} (V)	J_{SC} (mA•cm^{-2})	FF (%)	EQE Max.	ARC	Certified	Description and methods
9.1	0.701	20.64	62.5	1.00	With	Yes	Toyota Central R&D Lab., sputtering;[2]
8.4	0.661	19.5	65.8	0.88	With	Yes	IBM group, evaporation;[3; 4]
8.8	0.683	20.7	62.5	0.90	With	No	UNSW group, sputtering;[5]
8.76	0.666	19.47	67.51	0.85	With	No	UNSW group, sputtering;[6]
8.7	0.800	18.1	60.0	0.80	Without	No	Toyota Central R&D Lab., sputtering;[7]
7.0	0.691	16.2	62.4	0.75	Without	No	IBM group, stacking ED;[8]
7.4	0.675	17.36	63.13	0.80	Without	No	This work, alloy ED;

[a] PCE–power conversion efficiency; V_{OC}–open circuit voltage; J_{SC}–short circuit current density; FF–fill factor; ARC–antireflection coating; EQE Max.–the maximum value of the external quantum efficiency;

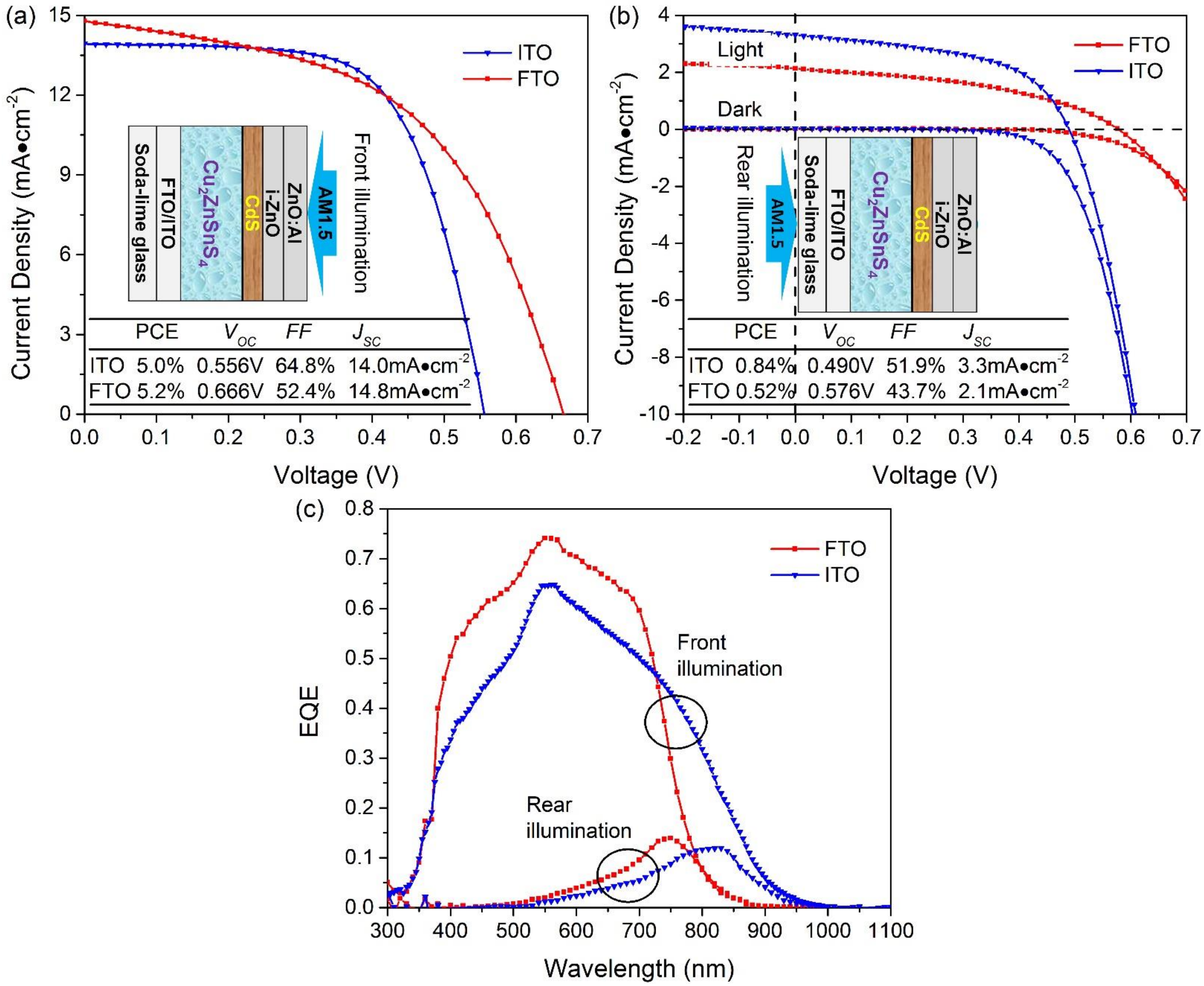

Figure S16. Current–voltage (J–V) characteristics and device parameters of finished glass/ITO or FTO/ CZTS /CdS/ZnO/AZO solar cells under the AM 1.5 global illumination from the AZO side (front illumination) (a) and from the ITO or FTO side (rear illumination) (b); spectral response of external quantum efficiency (EQE) curves under the front and rear illuminations, Related to **Figure 9**

Note: all the CZTS absorber films were sulfurized in the pure argon environment without hydrogen.

Supplemental References

1 Bär, M., Schubert, B.-A., Marsen, B., Krause, S., Pookpanratana, S., Unold, T., Weinhardt, L., Heske, C., and Schock, H.-W. (2011). Impact of KCN etching on the chemical and electronic surface structure of Cu_2ZnSnS_4 thin-film solar cell absorbers. Applied Physics Letters *99*, 152111.
2 Green, M.A., Emery, K., Hishikawa, Y., Warta, W., and Dunlop, E.D. (2015). Solar cell efficiency tables (version 46). Progress in Photovoltaics: Research and Applications *23*, 805-812.
3 Shin, B., Gunawan, O., Zhu, Y., Bojarczuk, N.A., Chey, S.J., and Guha, S. (2013). Thin film solar cell with 8.4% power conversion efficiency using an earth-abundant Cu_2ZnSnS_4 absorber. Progress in Photovoltaics: Research and Applications *21*, 72-76.
4 Green, M.A., Emery, K., Hishikawa, Y., Warta, W., and Dunlop, E.D. (2013). Solar cell efficiency tables (version 41). Progress in Photovoltaics: Research and Applications *21*, 1-11.
5 Yan, C., Sun, K., Huang, J., Johnston, S., Liu, F., Veettil, B.P., Sun, K., Pu, A., Zhou, F., Stride, J.A., *et al.* (2017). Beyond 11% efficient sulfide kesterite $Cu_2Zn_xCd_{1-x}SnS_4$ solar sell: Effects of cadmium alloying. ACS Energy Letters *2*, 930-936.
6 Liu, F., Yan, C., Huang, J., Sun, K., Zhou, F., Stride, J.A., Green, M.A., and Hao, X. (2016). Nanoscale microstructure and chemistry of Cu_2ZnSnS_4/CdS interface in kesterite Cu_2ZnSnS_4 solar cells. Advanced Energy Materials *6*, 1600706.
7 Tajima, S., Umehara, M., Hasegawa, M., Mise, T., and Itoh, T. (2017). Cu_2ZnSnS_4 photovoltaic cell with improved efficiency fabricated by high-temperature annealing after CdS buffer-layer deposition. Progress in Photovoltaics: Research and Applications *25*, 14-22.
8 Guo, L., Zhu, Y., Gunawan, O., Gokmen, T., Deline, V.R., Ahmed, S., Romankiw, L.T., and Deligianni, H. (2014). Electrodeposited $Cu_2ZnSnSe_4$ thin film solar cell with 7% power conversion efficiency. Progress in Photovoltaics: Research and Applications *22*, 58-68.
